# Antigen-Specific Adaptive Immunity to SARS-CoV-2 in Acute COVID-19 and Associations with Age and Disease Severity

**DOI:** 10.1016/j.cell.2020.09.038

**Published:** 2020-11-12

**Authors:** Carolyn Rydyznski Moderbacher, Sydney I. Ramirez, Jennifer M. Dan, Alba Grifoni, Kathryn M. Hastie, Daniela Weiskopf, Simon Belanger, Robert K. Abbott, Christina Kim, Jinyong Choi, Yu Kato, Eleanor G. Crotty, Cheryl Kim, Stephen A. Rawlings, Jose Mateus, Long Ping Victor Tse, April Frazier, Ralph Baric, Bjoern Peters, Jason Greenbaum, Erica Ollmann Saphire, Davey M. Smith, Alessandro Sette, Shane Crotty

**Affiliations:** 1 Center for Infectious Disease and Vaccine Research, La Jolla Institute for Immunology (LJI), La Jolla, CA 92037, USA; 2 Flow Cytometry Core Facility, La Jolla Institute for Immunology (LJI), La Jolla, CA 92037, USA; 3 Department of Medicine, Division of Infectious Diseases and Global Public Health, University of California, San Diego (UCSD), La Jolla, CA 92037, USA; 4 Department of Epidemiology, UNC Chapel Hill School of Public Health, University of North Carolina School of Medicine, Chapel Hill, NC 27599, USA; 5 Department of Microbiology and Immunology, University of North Carolina School of Medicine, Chapel Hill, NC 27599, USA

**Keywords:** coronavirus, CD4, CD8, T cells, antibody, neutralizing antibodies, adaptive immunity, CXCL10, IP-10, Spike, epitopes

## Abstract

Limited knowledge is available on the relationship between antigen-specific immune responses and COVID-19 disease severity. We completed a combined examination of all three branches of adaptive immunity at the level of SARS-CoV-2-specific CD4^+^ and CD8^+^ T cell and neutralizing antibody responses in acute and convalescent subjects. SARS-CoV-2-specific CD4^+^ and CD8^+^ T cells were each associated with milder disease. Coordinated SARS-CoV-2-specific adaptive immune responses were associated with milder disease, suggesting roles for both CD4^+^ and CD8^+^ T cells in protective immunity in COVID-19. Notably, coordination of SARS-CoV-2 antigen-specific responses was disrupted in individuals ≥ 65 years old. Scarcity of naive T cells was also associated with aging and poor disease outcomes. A parsimonious explanation is that coordinated CD4^+^ T cell, CD8^+^ T cell, and antibody responses are protective, but uncoordinated responses frequently fail to control disease, with a connection between aging and impaired adaptive immune responses to SARS-CoV-2.

## Highlights

• Adaptive immune responses limit COVID-19 disease severity
• Multiple coordinated arms of adaptive immunity control better than partial responses
• CXCL10 may be a biomarker of impaired T cell responses in acute COVID-19
• Aging and scarcity of naive T cells may be linked risk factors for severe COVID-19

## Graphical Abstract

**Figure:**
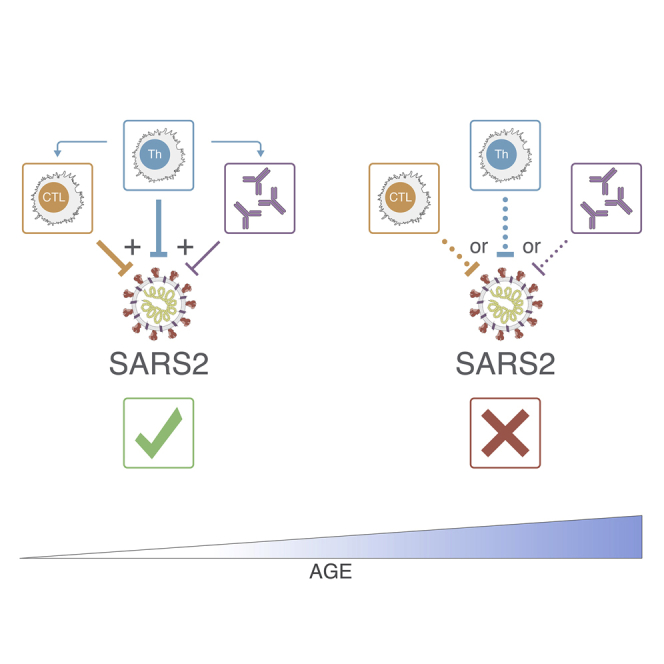

## Teaser

Analysis of SARS-CoV-2-specific adaptive immune responses during acute COVID-19 identifies coordination between SARS-CoV-2-specific CD4 T cells and CD8 T cells to limit disease severity. Aged individuals often exhibit uncoordinated adaptive responses, potentially tied to scarcity of naive T cells, highlighting immunologic risk factors linked to disease severity.

## Introduction

The ongoing COVID-19 pandemic has resulted in the infection of nearly 18 million people worldwide within 8 months, with over 4.5 million cases in the United States (World Health Organization). While most SARS-CoV-2 infections are not severe, a significant percentage of patients require hospitalization, and many fatalities occur, with increased rates of severe and fatal disease among older individuals (> 65 years old) (Docherty et al., 2020; Grasselli et al., 2020) and those with pre-existing medical conditions like cardiovascular disease, obesity, and type 2 diabetes mellitus (Docherty et al., 2020; Richardson et al., 2020). Severe cases can progress to respiratory failure associated with diffuse alveolar damage and acute respiratory distress syndrome (ARDS) (Grasselli et al., 2020; Richardson et al., 2020), similar to what was observed for SARS (Rockx et al., 2020). The relative role(s) played by the immune response to SARS-CoV-2 versus direct viral effects in the respiratory system and other organ systems has been questioned, with the possibility of immunopathogenesis being a major causal component of severe COVID-19 (McKechnie and Blish, 2020; Vabret et al., 2020). Elevated innate immune cytokines detected in peripheral blood including interleukin (IL)-1, IL-6, IL-8, or C-X-C Motif Chemokine Ligand 10 (CXCL10) have been associated with severe or fatal COVID-19 (Blanco-Melo et al., 2020; Laing et al., 2020; Lucas et al., 2020; Vabret et al., 2020; Del Valle et al., 2020). However, insufficient information directly examining SARS-CoV-2 antigen-specific CD4^+^ T cells, CD8^+^ T cells, and neutralizing antibodies in the same acute patients is hindering our understanding of the roles of adaptive immunity in acute COVID-19 protection or pathogenesis. SARS-CoV-2 antigen-specific adaptive immune responses (ADIM) have been inferred from surrogate markers in large studies (Laing et al., 2020; Lucas et al., 2020; Mathew et al., 2020), and some antigen-specific T cell (Meckiff et al., 2020; Weiskopf et al., 2020) or neutralizing antibody data (Robbiani et al., 2020; Rogers et al., 2020; Wajnberg et al., 2020) are available, but combined assessments of antigen-specific CD4^+^ T cells, CD8^+^ T cells, and neutralizing antibodies in acute COVID-19 are still lacking (except 3 subjects in Zhou et al., 2020b). Addressing these fundamental questions is important for the clinical management of COVID-19, as well as for proper COVID-19 vaccine development cognizant of protective immune responses and potential immunopathogenic responses.

The adaptive immune system responds to pathogens in an antigen-specific manner to develop protective immunity. The adaptive immune system consists of three major lymphocyte types: B cells (antibody producing cells), CD4^+^ T cells (helper T cells), and CD8^+^ T cells (cytotoxic, or killer, T cells) (Murphy and Weaver, 2016). All three arms of adaptive immunity can be important in protection against viral infections. The vast majority of licensed human vaccines work on the basis of protective antibody responses, with neutralizing antibodies being the most common mechanism of action (Piot et al., 2019; Plotkin, 2010; Plotkin et al., 2018). Thus, most COVID-19 vaccine efforts focus on the elicitation of neutralizing antibodies (Amanat and Krammer, 2020; Corey et al., 2020; Thanh Le et al., 2020), with additional interest in elicitation of CD4^+^ or CD8^+^ T cells (Corbett et al., 2020; Folegatti et al., 2020; Jackson et al., 2020; Mercado et al., 2020; Sahin et al., 2020). Almost all neutralizing antibody responses, durable antibody responses, and affinity-matured B cell memory depend on CD4^+^ T cell help (Crotty, 2019). As such, CD4^+^ T cell responses are critical to the success of most vaccines. Additionally, CD4^+^ T cells have a range of different functionalities beyond helping antibody responses that can be valuable in the context of antiviral immunity (Zhu et al., 2010). In a mouse model of SARS, it was demonstrated that CD4^+^ T cells alone, in the absence of antibodies or CD8^+^ T cells, could provide protection against lethal challenge with SARS-CoV (Zhao et al., 2016). Separately, extensive animal model studies have proven the importance of CD8^+^ T cells in protective immunity against a range of viral infections (Chang et al., 2014; Masopust and Soerens, 2019). Thus, it is important to assess all three arms of adaptive immunity in SARS-CoV-2-infected individuals across the spectrum of COVID-19 disease severity in a coordinated manner to gain insights into SARS-CoV-2 protective immunity and potential immunopathogenesis.

## Results

**Figure S1:**
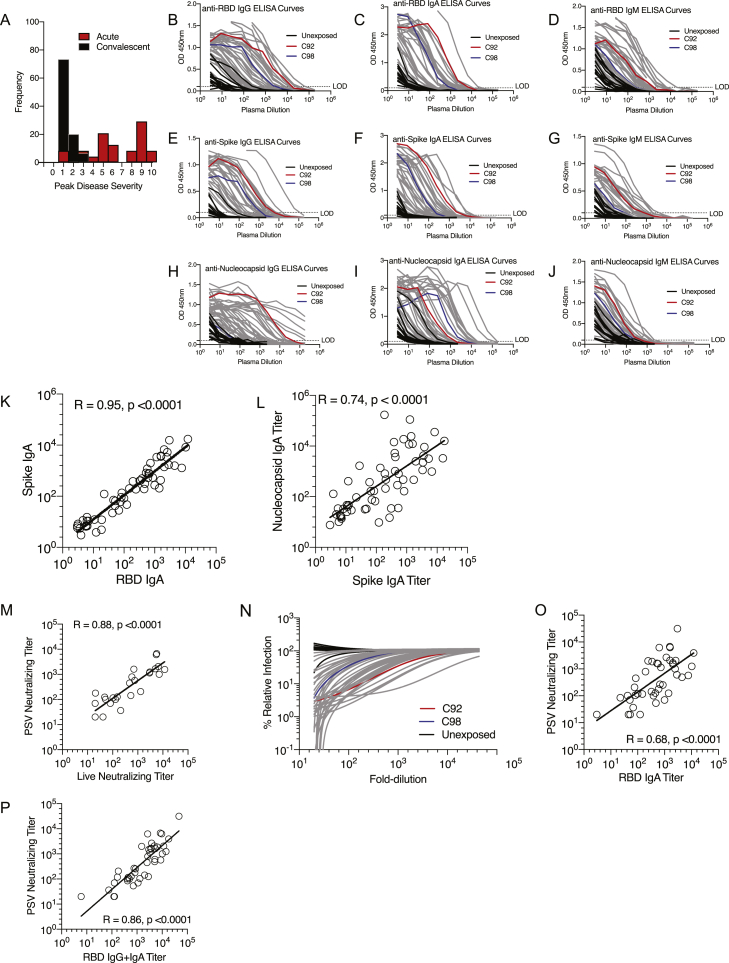
SARS-CoV-2 Antibody Responses, Related to Figure 1 (A) Frequency of Peak Disease Severity (1-10) for acute and convalescent COVID-19. (B-J) Plasma ELISA curves for SARS-CoV-2 spike RBD (B) IgG, (C) IgA, (D) IgM; SARS-CoV-2 Spike (E) IgG, (F) IgA, (G) IgM; and SARS-CoV-2 Nucleocapsid (N) protein (H) IgG, (I) IgA, and (J) IgM. C92 and C98 are representative acute donors. Grey lines = all other COVID-19 samples. The dotted line indicates LOD. (K) Correlation of Spike IgA with RBD IgA. (L) Correlation of N IgA with Spike IgA. (M) Correlation of PSV neutralizing titer with live neutralizing titer. (N) PSV titration curves. C92 and C98 are representative acute COVID-19 donors. Grey lines = all other COVID-19 samples. (O, P) PSV neutralizing titer correlates with RBD IgA (O) and RBD IgG+IgA (P). In (K-M) and (O-P), white dots = all COVID-19 (acute and convalescent). Statistics reported for all COVID-19 cases.

We set out to measure fundamental metrics of all three arms of the antigen-specific adaptive immune responses (ADIMs) to SARS-CoV-2 and then relate those antigen-specific immune responses to COVID-19 disease severity in acutely ill and convalescent individuals. In our earlier work, we measured CD4^+^ and CD8^+^ T cell responses in a cohort of average, non-hospitalized cases of COVID-19 during the convalescent phase as a first benchmark of ADIMs to SARS-CoV-2 (Grifoni et al., 2020a). That study did not include acute patients and did not include measurement of SARS-CoV-2-neutralizing antibodies. Here, we measured SARS-CoV-2-specific antibodies (including neutralizing antibodies), SARS-CoV-2-specific CD4^+^ T cells, and SARS-CoV-2-specific CD8^+^ T cells in all individuals in a new cohort, with an emphasis on including acute cases across a range of COVID-19 disease severities. 54 subjects were enrolled in this study, 24 subjects with acute COVID-19. Maximum disease severity ranged from mild to fatal (Table S1; Figure S1A). Days post-symptom onset (PSO) for sample collection ranged from d4-37 (Table S1). We also obtained a second or third blood sample from 5 subjects with acute COVID-19. Fifteen convalescent subjects and 15 unexposed control subjects were also enrolled (Table S1). SARS-CoV-2-specific antibodies, CD4^+^ T cells, and CD8^+^ T cells were each quantified by multiple methods.

### SARS-CoV-2-Specific Antibody Responses

**Figure 1:**
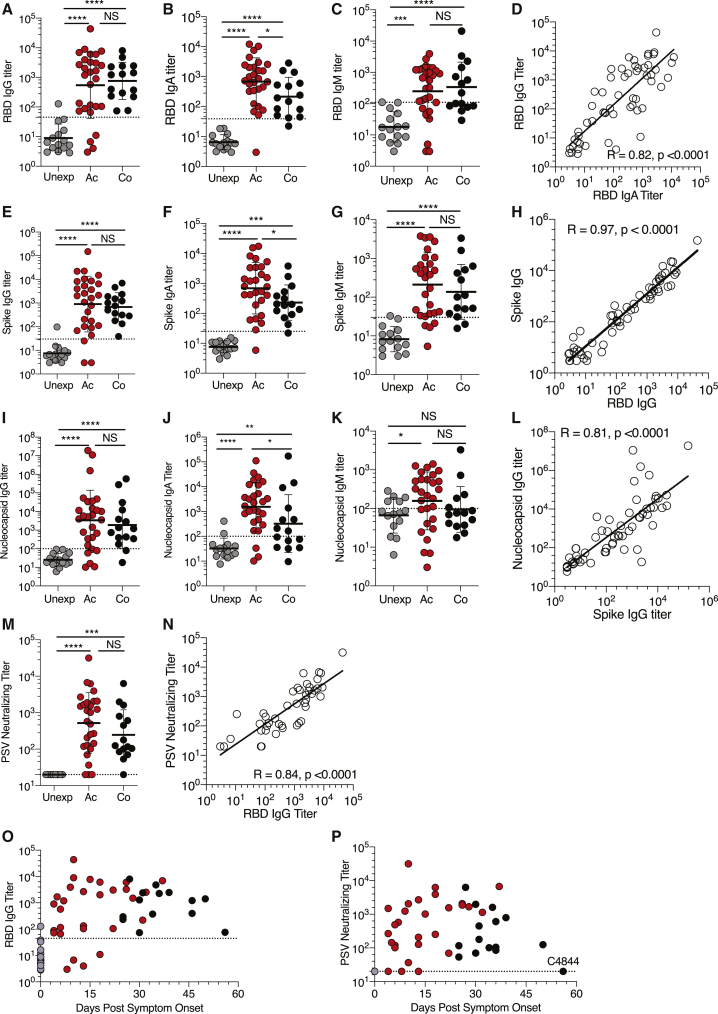
SARS-CoV-2 Antibody Responses in COVID-19 Subjects (A–C) Plasma antibody titers to SARS-CoV-2 S RBD (A) IgG, (B) IgA, and (C) IgM, divided into unexposed n = 15, acute (Ac) n = 28, and convalescent (Co) n = 15. (D) SARS-CoV-2 RBD IgG correlates with RBD IgA. (E–G) Plasma ELISA titers to SARS-CoV-2 S (E) IgG, (F) IgA, and (G) IgM. (H) S IgG correlation with RBD IgG. (I–K) Plasma ELISA titers to SARS-CoV-2 Nucleocapsid (N) protein (I) IgG, (J) IgA, and (K) IgM. (L) N IgG correlation with S IgG. (M) Pseudovirus (PSV) neutralizing antibody titers in unexposed, acute, and convalescent COVID-19 samples. (N) PSV neutralizing antibody titers correlated with RBD IgG titers. (O and P) Both (O) RBD IgG and (P) PSV neutralizing Ab titers were detectable in most acute and all convalescent COVID-19 cases at all time points tested. The dotted line indicates LOD. Geometric mean titers with geometric SDs are indicated. Acute (Ac) = Red, Convalescent (Co) = black, Unexposed = gray. White = all COVID-19 (acute and convalescent). ^∗^p < 0.05, ^∗∗^p < 0.01, ^∗∗∗^p < 0.001, ^∗∗∗∗^p < 0.0001, NS = not significant. See also Figure S1.

The RBD domain of SARS-CoV-2 Spike (S) is highly divergent from other CoVs (Premkumar et al., 2020). The RBD domain is the primary target of SARS-CoV-2-neutralizing antibodies (Ju et al., 2020; Rogers et al., 2020), much like what was found for SARS-CoV (Subbarao, 2020). Therefore, we measured SARS-CoV-2 RBD immunoglobulin (Ig) G, IgM, and IgA titers in all subjects. RBD IgG was detectable in almost all COVID-19 cases (24/28 acute, 15/15 convalescent; Figures 1A and S1B), although 28% of cases had relatively low titers (within 3-fold of the limit of detection, LOD). RBD IgA was also consistently detected (41/43; Figures 1B and S1C) and correlated well with RBD IgG (Figure 1D). Distinguishable RBD IgM was observed less often (Figures 1C and S1D) for both acute and convalescent cases, consistent with other recent reports (Robbiani et al., 2020). Full-length SARS-CoV-2 S IgG, IgM, and IgA titers were also measured. S IgG and IgA responses were robust in most COVID-19 cases (25/28 acute, 15/15 convalescent S IgG; Figures 1E and S1E) (27/28 acute, 14/15 convalescent S IgA; Figures 1F and S1F), though ∼17% of cases had relatively low titers (within 3-fold of the LOD). Similar to RBD IgM, S IgM was less frequently observed (Figures 1G and S1G). S IgG & IgA titers correlated with RBD IgG (Figure 1H) and IgA titers (Figure S1K). Ig titers were also measured against SARS-CoV-2 Nucleocapsid (N), as that antigen is frequently used in serodiagnostic assays (Okba et al., 2020). N IgG and IgA were detected in most COVID-19 cases (Figures 1I, 1J, S1H, and S1I). N IgM was detected in fewer COVID-19 cases (Figures 1K and S1J). S IgG and IgA titers partially correlated with N IgG and IgA (Figures 1L and S1L).

In addition to quantifying SARS-CoV-2-binding antibodies, we measured functional SARS-CoV-2 antibodies by neutralization assays (Figures S1M and S1N). Performance of a live virus neutralization assay and pseudovirus (PSV) neutralization assay was comparable (r = 0.88, p < 0.0001, two-sided Spearman rank-correlation test) (Figures S1M and S1N), and thus the majority of the data were subsequently obtained with the PSV neutralization assay. SARS-CoV-2-neutralizing antibodies were detectable in almost all COVID-19 cases (25/28 acute, 14/15 convalescent; Figure 1M). These results are similar to those reported in other studies using neutralization assays with similar LOD (Suthar et al., 2020; Wu, 2020). SARS-CoV-2-neutralizing antibody titers correlated with RBD IgG and RBD IgA (Figures 1N, S1O, and S1P), consistent with findings that RBD is the primary target of SARS-CoV-2-neutralizing antibodies in humans. Neutralizing antibodies and RBD IgG were detectable in the majority of patients in all time windows (Figures 1O and 1P) (Suthar et al., 2020). Overall, this battery of serological assays found that most acute and convalescent COVID-19 case subjects had detectable circulating antibodies against SARS-CoV-2 RBD, S, and N, as well as neutralizing antibodies.

### SARS-CoV-2-Specific CD4^+^ T Cell Responses

**Figure 2:**
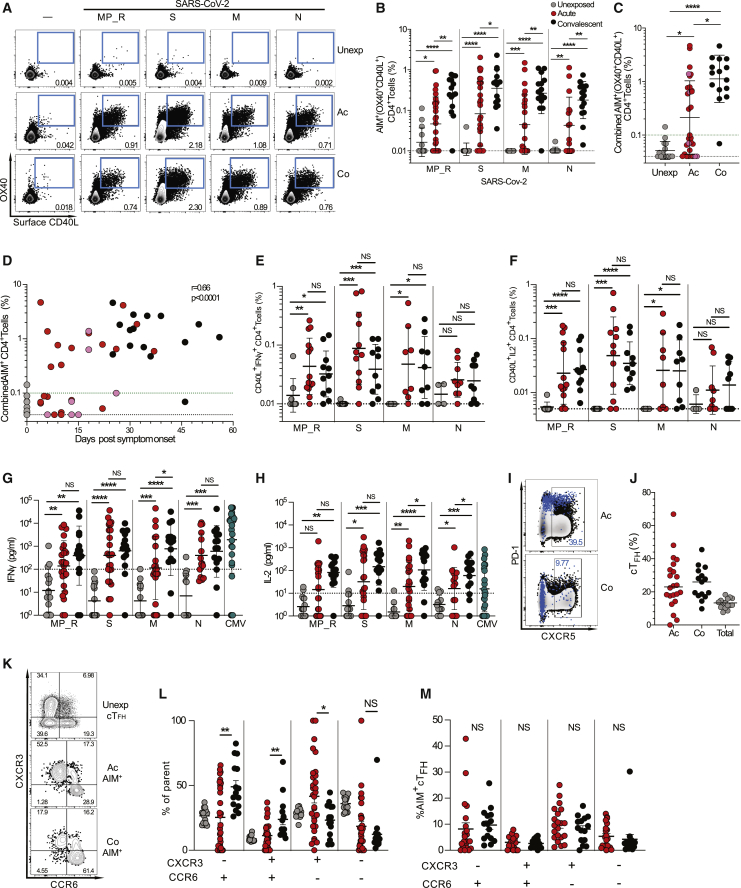
SARS-CoV-2-Specific CD4^+^ T Cell Responses (A) Representative flow cytometry gating of AIM^+^ (OX40^+^surfaceCD40L^+^) CD4^+^ T cells. (B) Percentage of background subtracted SARS-CoV-2-specific total CD4^+^ T cells quantified by AIM after stimulation with MP_R (Non-Spike), S (Spike), M (Membrane), or N (Nucleocapsid) peptide pools in unexposed (n = 15), acute COVID-19 (n = 30) and convalescent COVID-19 (n = 15). (C and D) Percentage of background subtracted combined MP_R, S, M, and N SARS-CoV-2-specific CD4^+^ T cells by AIM assay by (C) cohort and (D) by days PSO. Combined AIM responses were calculated as the sum of the CD4^+^ AIM response to background-subtracted individual peptide megapools. Statistics in (D) are reported for unexposed, convalescent, and acute samples. (E and F) ICS of SARS-CoV-2-specific CD4^+^ T cells quantified by co-expression of (E) CD40L^+^IFNγ^+^ or (F) CD40L^+^IL-2^+^ after stimulation with SARS-CoV-2 peptide pools in unexposed (n = 8), acute COVID-19 (n = 14) and convalescent COVID-19 samples (n = 11). (G and H) Cytokines IFNγ (G) and IL-2 (H) in the supernatants after stimulation with SARS-CoV-2 or CMV peptide pools in unexposed (n = 15), acute COVID-19 (n = 22), convalescent COVID-19 (n = 15), and CMV^+^ controls (n = 23). The black dotted line delineates background signal as determined by the unexposed controls. (I) Representative flow cytometry of SARS-CoV-2-specific (OX40^+^CD40L^+^) CD4^+^ T cells (blue dots) overlaid on total CD4^+^T cells (black dots). (J) Percentage of SARS-CoV-2-specific cT_FH_ cells in acute COVID-19 (n = 22) or convalescent COVID-19 (n = 15) samples that had a positive total CD4^+^ AIM response (> 0.04%) following stimulation with the SARS-CoV-2 S megapool (MP), or the total non-antigen-specific CXCR5^+^ CD4^+^ T cells in unexposed controls (n = 15, gray dots) (median displayed). (K) Representative fluorescence-activated cell sorting (FACS) plots of CXCR3 and CCR6 staining in total cT_FH_ (CXCR5^+^CD4^+^ cells) in unexposed donors or S-specific AIM^+^ (OX40^+^CD40L^+^) CD4^+^ T cells in acute and convalescent donors. (L and M) Frequency of (L) CXCR3 and/or CCR6 expressing S-specific AIM^+^ cells out of total CD4^+^ T cells in acute or convalescent samples or non-antigen-specific CXCR5^+^CD4^+^ cT_FH_ in unexposed samples (n = 15) and (M) CXCR5^+^ S-reactive AIM^+^ cells out of total CD4^+^ T cells in acute donors (n = 26 samples) or convalescent donors (n = 15 samples). Unless otherwise stated, the black dotted line indicates LOD; the green dotted line demarcates marginal responses as determined by unexposed donor responses. Pink dots denote samples where two or more peptide pools were not run due to cell numbers. ^∗^p < 0.05, ^∗∗^p < 0.01, ^∗∗∗^p < 0.001, ^∗∗∗∗^p < 0.0001, NS = not significant. See also Figure S2, Table S4, and Table S5.

**Figure S2:**
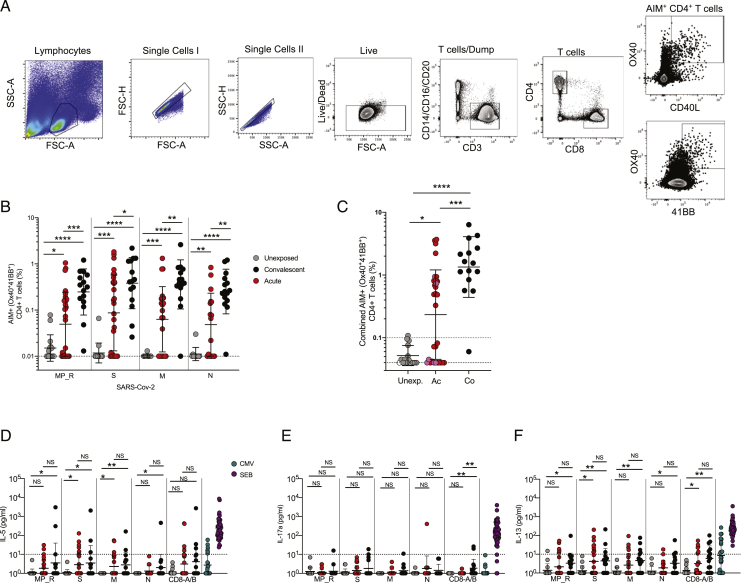
SARS-CoV-2-Specific CD4^+^ T Cell Responses, Related to Figure 2 (A) Gating strategy for identification of SARS-CoV-2-antigen-specific CD4^+^ T cells. (B) % of OX40^+^4-1BB^+^ CD4^+^ T cells specific for SARS-CoV-2 MP_R, S, M, N peptide megapools by AIM (C) Combined % Ox40^+^4-1-BB^+^ CD4^+^ T cells across all SARS-CoV-2 peptide megapools by AIM. The black dotted line indicates LOD; the green dotted line demarcates marginal responses as determined by unexposed donor responses. Pink dots denote samples where two or more peptide pools were not run due to cell numbers. (D-F) Amount (pg/mL) of (D) IL-5 (E), IL-13 (F), and IL-17 in the AIM supernatants after stimulation with MP_R, S, M, N, and CMV peptide pools. The black dotted line delineates background signal as determined by the unexposed controls. Acute (Ac) = Red, Convalescent (Co) = black, Unexposed (Unexp) = gray. ^∗^p < 0.05, ^∗∗^p < 0.01, NS = not significant.

SARS-CoV-2-specific CD4^+^ T cells were measured using *in vitro* stimulation with SARS-CoV-2 peptide pools followed by quantitation of antigen-specific cells in a cytokine agnostic fashion by T cell receptor (TCR) activation-induced markers (AIM, surface CD40L^+^OX40^+^) (Grifoni et al., 2020a; Morou et al., 2019; Reiss et al., 2017) in live cell flow cytometry, using peripheral blood mononuclear cell (PBMC) samples from all subjects. CD4^+^ T cells specific for major antigens S, N, and membrane (M) were measured directly with overlapping peptides covering each full protein sequence. Additionally, a “megapool” (MP) of peptides representing the top predicted human leukocyte antigen **(**HLA) class II epitopes outside of S was used to measure CD4^+^ T cells directed against the remainder of the SARS-CoV-2 orfeome MP (megapool remainder, MP_R; Figures 2A and 2B) (Grifoni et al., 2020b, 2020a). A cumulative SARS-CoV-2-specific CD4^+^ T cell measurement was calculated as the sum of the S-, N-, M-, and MP_R-specific CD4^+^ T cells (Figure 2C). SARS-CoV-2-specific CD4^+^ T cells were detected in almost all convalescent COVID-19 samples by AIM (14/15; Figure 2C), with consistent responses against S, M, N, and MP_R (Figure 2A), matching our previous cohort of convalescent COVID-19 cases (Grifoni et al., 2020a). However, SARS-CoV-2-specific CD4^+^ T cells were detected in only 77% of acute COVID-19 samples (23/30) (Figure 2C), with similar observations for individual peptide pools (S, M, N, and MP_R; Figures 2A and 2B). Furthermore, 27% of responses were borderline or weak CD4^+^ T cell responses (8/30. Defined as > LOD [0.04%] but < 0.1% SARS-CoV-2-specific combined CD4^+^ cells. Figure 2C). Results were comparable using alternative AIM markers (OX40^+^CD137/41BB^+^; Figures S2A–S2C). SARS-CoV-2-specific CD4^+^ T cells were detected as early as d4 PSO (Figure 2D). Overall, robust levels of circulating SARS-CoV-2-specific CD4^+^ T cells were only detected in 50% of acute COVID-19 samples (15/30), in contrast to 93% of samples in convalescent cases (14/15, > 0.1% SARS-CoV-2-specific combined CD4^+^ cells). To the extent that cell number availability allowed, intracellular cytokine staining was performed as an independent measurement of SARS-CoV-2-specific CD4^+^ T cells, using the S, N, M, and MP_R peptides (Figures 2E and 2F). Interferon gamma (IFNγ) and IL-2 were detected by ICS in both acute and convalescent COVID-19 cases, consistent with cytokine measurements from peptide-stimulated supernatants (Figures 2G and 2H). Minimal to no IL-5, IL-13, or IL-17a secretion was detected from SARS-CoV-2-specific T cells from acute or convalescent samples (Figures S2D–S2F), similar to that of CMV-specific T cells (Figures S2D–S2F). Non-T follicular helper (T_FH_) CD4^+^ T cells in antiviral immune responses usually predominantly consist of type I T helper (T_H_1) cells, which can have direct antiviral functions, recruit monocytes to infected tissues, or help CD8^+^ T cells. IFNγ and IL-2 were the primary secreted cytokines detected after SARS-CoV-2 peptide stimulation for both acute and convalescent cases (Figures 2G and 2H).

Circulating T follicular helper (cT_FH_) and non-T_FH_ cells specific for SARS-CoV-2 could be distinguished by CXCR5 expression. T_FH_ cells are the CD4^+^ T cells required for most IgG responses and high-quality neutralizing antibodies (Crotty, 2019). Virus-specific cT_FH_ cells were a substantial fraction of the SARS-CoV-2-specific CD4^+^ T cells in acute and convalescent COVID-19 cases (Figures 2I and 2J). Additionally, CXCR3 and chemokine receptor 6 (CCR6) were expressed on subpopulations of SARS-CoV-2-specific CD4^+^ T cells (Figures 2L and 2M). Among SARS-CoV-2-specific cT_FH_ cells (CXCR5^+^ AIM^+^), CXCR3^+^CCR6^-^, CXCR3^-^CCR6^+^, CXCR3^+^CCR6^+^, and CXCR3^-^CCR6^-^ cells were observed (Figure 2M). S-specific CXCR3^-^CCR6^+^ cT_FH_ were also observed in a study of convalescent COVID-19 cases (Juno et al., 2020). IL-17a expression by the SARS-CoV-2-specific CD4^+^ T cells was generally not observed (Figure S2F), suggesting that the CCR6 expression by these CD4^+^ T cells may be primarily an indicator of lung-homing. Overall, the CD4^+^ T cell response in acute COVID-19 cases largely consisted of T_FH_ cells and IFNγ-producing cells, consistent with proper antiviral polarization.

### SARS-CoV-2-Specific CD8^+^ T Cell Responses

**Figure 3:**
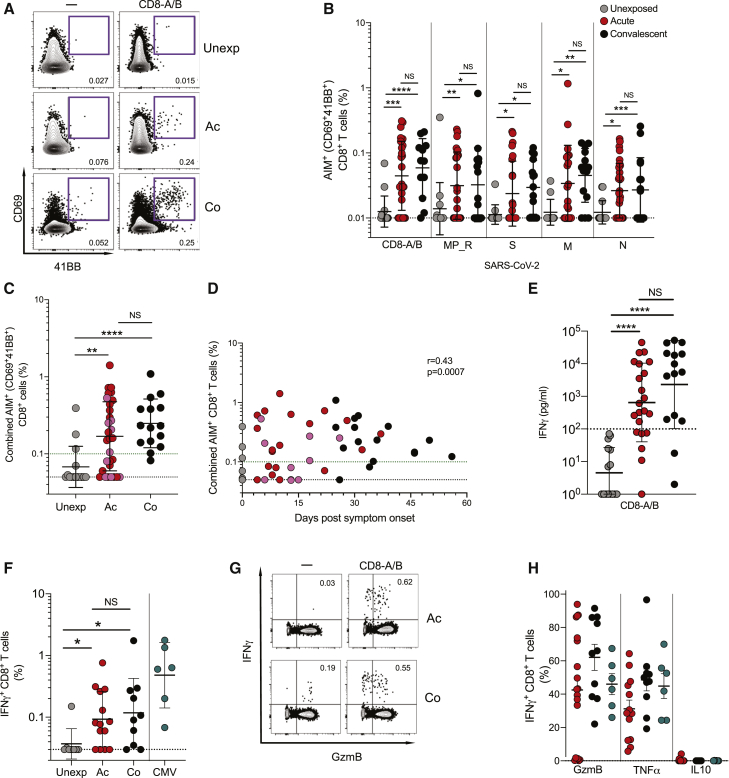
SARS-CoV-2-Specific CD8^+^ T Cell Responses (A) Sample flow cytometry gating of AIM (CD69^+^4-1BB^+^) CD8^+^ T cells. (B) Percentage of background subtracted SARS-CoV-2-specific total CD8^+^ T cells via AIM assay after stimulation with CD8_A/B, MP_R (Non-Spike), S (Spike), M (Membrane), N (Nucleocapsid) peptide pools in unexposed (n = 15), acute COVID-19 (n = 30) and convalescent COVID-19 (n = 15). (C and D) Percentage of background subtracted combined CD8-A/B, R, S, M, and N SARS-CoV-2-specific total CD4^+^ T cells by AIM assay (C) by cohort and (D) by days PSO. Combined AIM responses were calculated as the sum of the CD8^+^ AIM response to background-subtracted individual peptide megapools. Statistics in (D) are reported for unexposed, acute, and convalescent samples. (E) Quantitation of IFNγ in supernatants after stimulation with peptide pools unexposed (n = 15), acute COVID-19 (n = 21), and convalescent COVID-19 (n = 15). The black dotted line delineates background signal as determined by the unexposed controls. (F) Percentage of background subtracted SARS-CoV-2-specific total CD8^+^ T cells quantified by expression of IFNγ^+^ by ICS. (G) Representative flow cytometry gating of IFNγ^+^GzmB^+^ CD8^+^ T cells in acute and convalescent COVID-19 samples. (H) Percentage of IFNγ^+^ CD8^+^ T cells expressing granzyme B (GzmB), TNFα, or IL10 by ICS in unexposed (n = 8), acute COVID-19 (n = 14) and convalescent COVID-19 (n = 11). Unless otherwise stated, the black dotted line indicates LOD; the green dotted line demarcates marginal responses as determined by unexposed donor responses. Pink dots denote samples where two or more peptide pools were not run due to cell numbers. ^∗^p < 0.05, ^∗∗^p < 0.01, ^∗∗∗^p < 0.001, ^∗∗∗∗^p < 0.0001, NS = not significant. See also Figure S3, Table S4, and Table S5.

**Figure S3:**
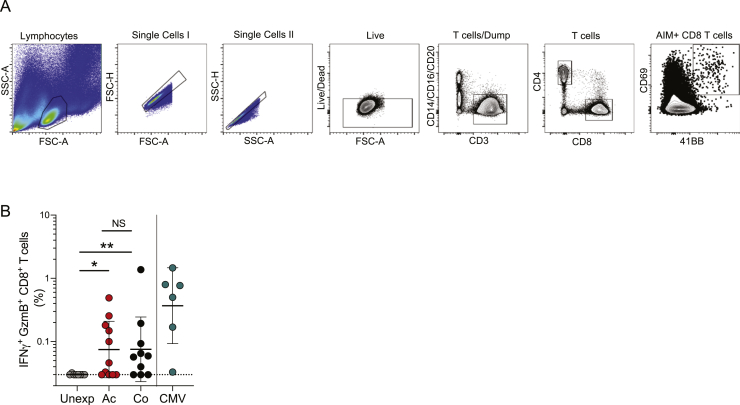
SARS-CoV-2-Specific CD8+ T Cell Responses, Related to Figure 3 (A) Gating strategy for identification of SARS-CoV-2-antigen-specific CD8^+^ T cells. (B) Percentage of background subtracted SARS-CoV-2-specific total CD8^+^ T cells quantified by co-expression of IFNg and granzyme B (GzmB) by ICS in unexposed (n = 8), acute COVID-19 (n = 11) and convalescent COVID-19 (n = 11). The black dotted line indicates LOD. Acute (Ac) = Red, Convalescent (Co) = black, Unexposed (Unexp) = gray. ^∗^p < 0.05, ^∗∗^p < 0.01. NS = not significant.

SARS-CoV-2-specific CD8^+^ T cells were measured using *in vitro* stimulation with SARS-CoV-2 peptide pools followed by AIM flow cytometry (surface CD69^+^CD137/4-1BB^+^; Figures 3A and 3B). Two megapools of peptides representing the top predicted HLA class I SARS-CoV-2 epitopes (CD8-A, CD8-B) (Grifoni et al., 2020a) were used, and the results were combined. CD8^+^ T cells specific for S, N, M, and MP_R were also measured (Figures 3A and 3B). A cumulative SARS-CoV-2-specific CD8^+^ T cell measurement was calculated (Figure 3C). SARS-CoV-2-specific CD8^+^ T cells were detected in 87% of convalescent COVID-19 samples (13/15, > 0.1% combined AIM^+^ CD8^+^ T cells; Figure 3C). However, SARS-CoV-2-specific CD8^+^ T cells were detected in only 53% of acute COVID-19 samples (16/30; Figure 3C). SARS-CoV-2-specific CD8^+^ T cells were detected as early as d4 PSO (Figure 3D). Intracellular cytokine staining (ICS) was performed as an independent measurement of SARS-CoV-2-specific CD8^+^ T cells, in parallel with quantitation of cytokines secreted into the medium during the AIM T cell stimulation assays (Figures 3E–3H). The majority of acute and convalescent COVID-19 samples had measurable IFNγ^+^ CD8^+^ T cell responses by both ICS and secreted cytokines (Figures 3E–H and S3B). SARS-CoV-2-specific IFNγ^+^ CD8^+^ T cells predominantly expressed granzyme B, with detectable tumor necrosis factor alpha (TNFα) and absence of IL-10, with a functional profile comparable to that of cytomegalovirus (CMV)-specific CD8 T cells (Figure 3H).

### Relationships between ADIMs and COVID-19 Disease Severity

**Figure 4:**
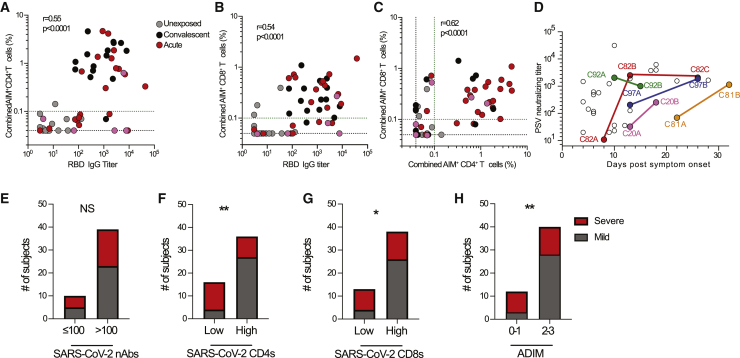
Coordinated Adaptive Immune Responses to SARS-CoV-2 (A) Correlation of SARS-CoV-2-specific CD4^+^ T cells and RBD IgG. (B) Correlation of SARS-CoV-2-specific CD8^+^ T cells and RBD IgG. (C) Correlation of SARS-CoV-2-specific CD4^+^ and CD8^+^ T cells. (D) PSV neutralizing antibody titers over time for acute COVID-19 subjects with paired blood samples. Open circles denote other acute COVID-19 samples. Unexposed controls (n = 15), acute COVID-19 (n = 26), and convalescent COVID-19 (n = 15). The black dotted line indicates LOD; the green dotted line demarcates marginal responses as determined by unexposed donor responses. Pink dots denote samples where two or more peptide pools were not run due to cell numbers. (E) Association between SARS-CoV-2 PSV-neutralizing antibodies and peak disease severity. (F and G) Association between (F) SARS-CoV-2-specific CD4^+^ T cells and (G) SARS-CoV-2-specific CD8^+^ T cells (“Low” < 0.1%, “High” > 0.1% combined AIM^+^) and COVID-19 peak disease severity. (H) Association between ADIM score and COVID-19 peak disease severity. Acute COVID-19 samples (n = 26) and convalescent COVID-19 samples (n = 26). Statistics for (A–C) are reported for unexposed, convalescent, and acute samples. ^∗^p < 0.05, ^∗∗^p < 0.01, NS = not significant. See also Figure S4 and Data S1.

**Figure S4:**
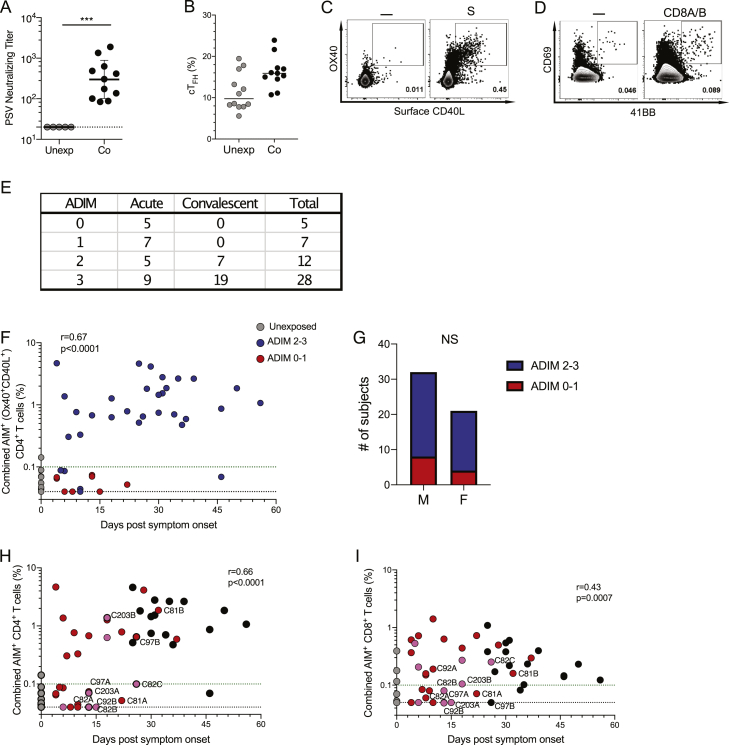
Coordinated Adaptive Immune Responses, Related to Figure 4 (A-B) PSV neutralizing titer (A) and (B) percentage of SARS-CoV-2-specific T_FH_ cells in samples that had a positive total CD4^+^ AIM response (> 0.04%) following stimulation with the SARS-CoV-2 S MP, or the total CXCR5^+^ CD4^+^ T cells in unexposed controls in additional unexposed donors (n = 12) and convalescent COVID-19 donors (n = 11). (C-D) Flow cytometry of AIM^+^ CD4^+^ T cell response (C) and AIM+ CD8^+^ T cells response (D) in donor C4844. (E) Frequency of ADIM observed in the cohort. (F) Correlation of SARS-CoV-2-specific total CD4^+^ T cells by OX40^+^CD40L^+^ AIM assay and days PSO, stratified into Immunotypes. Statistics are reported for unexposed, convalescent and acute samples. (G) Frequency of ADIM by gender. (H-I) Combined CD4^+^ AIM data based on day PSO from Fig. 2D and 3D with responses labeled for specific donors of interest. Statistics in (H-I) are reported for unexposed, convalescent and acute samples. The black dotted line indicates LOD; the green dotted line demarcates marginal responses as determined by unexposed donor responses. Pink dots denote samples where two or more peptide pools were not run due to cell numbers. NS = not significant, ^∗∗∗^p < 0.001, geometric mean with geometric SD displayed in S4A, median displayed in S4B.

The antigen-specific ADIM data from all COVID-19 cases were then compiled and examined together. First, by pairwise comparisons including all acute and convalescent cases, the SARS-CoV-2 antigen-specific antibody, CD4^+^ T cell, and CD8^+^ T cell immune responses exhibited positive correlations (p < 0.0001; Figures 4A–4C). The clinical environment and cell number requirements for antigen-specific T cell assays precluded acquisition of serial samples in most cases. Nevertheless, paired serial samples were obtained for five acute COVID-19 cases among the cohort (Figure 4D). Notably, of the four individuals who were the slowest to develop neutralizing antibody titers > 200, three of the four developed severe COVID-19 before they mounted a strong neutralizing antibody response (C97, C203, C81). Patient C97 had peak disease severity of 9 by d14 PSO, but only developed a neutralizing antibody titer of 1,000 at d26 PSO (Figure 4D). Most dramatically, patient C81 had peak COVID-19 severity 9 while neutralizing antibody titers were below 100 (potentially undetectable, as no C81 sample was available during peak severity d15 PSO). Patient C81 still had a marginal neutralizing antibody titer of <100 at d22 PSO and only developed a high neutralizing antibody titer at d32, which was three weeks slower than many COVID-19 cases (Figure 4D). Examining the T cell responses of these patients, C97 had COVID-19 severity 9 without detectable antiviral CD4^+^ T cell or CD8^+^ T cell responses at day 13 PSO (Figures 2D, 3D, S4H, and S4I). C81 provided yet another example, even more extreme, with SARS-CoV-2-specific CD4^+^ and CD8^+^ T cells below 0.1% as late as d22 PSO, only becoming > 0.1% at d32 PSO (Figures 2D, 3D, S4H, and S4I). C92 had a high neutralizing antibody titer (∼2000) at d10 PSO while having COVID-19 severity 9 and succumbed to disease at d26. Notably, C92 had undetectable SARS-CoV-2-specific CD4^+^ T cells at both d10 and d15 and undetectable SARS-CoV-2-specific CD8^+^ T cells at d15 PSO (Figures 1T, 2D, 3D, S4H, and S4I). Thus, fatal COVID-19 case C92 represented an uncoordinated ADIM, with neutralizing antibodies but a largely undetectable SARS-CoV-2-specific CD4^+^ T cell and CD8^+^ T cell response.

**Figure S5:**
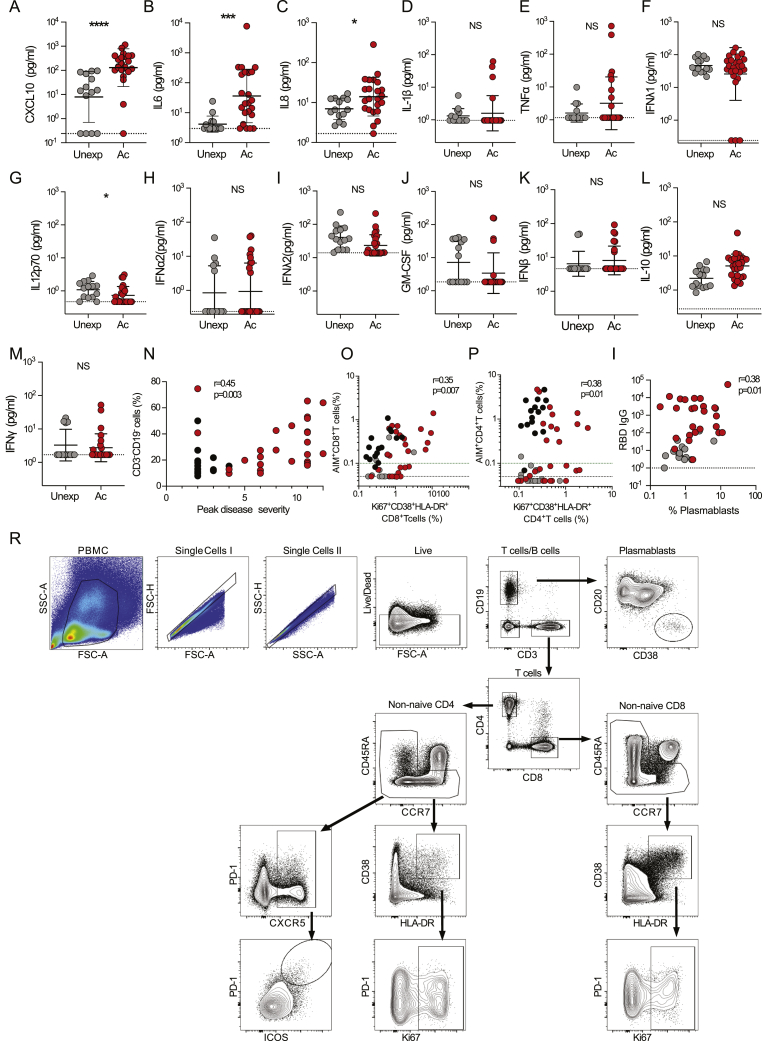
Plasma Cytokines and Immunophenotyping, Related to Figure 5 (A-M) Quantitation of plasma cytokine levels from acute (n = 24) and unexposed (n = 15) donors: (A) CXCL10, (B) IL-8, and (C) IL-6. (D) IL-1b, (E) TNFa, (F) IFNl1, (G) IL12p70, (H) IFNa2, (I) IFNl2, (J) GM-CSF, (K) IFNb, (L) IL-10, (M) IFNg. (N) Correlation of CD3^-^CD19^-^ PBMCs and peak disease severity. Statistics are reported for convalescent and acute samples. (O-P) Correlation of (O) activated CD4 T cells with combined AIM+ CD4 T cells and (P) activated CD8 T cells with combined AIM+ CD8 T cells across all donors. Statistics are reported for unexposed, convalescent and acute samples. (Q) Correlation of (CD38^hi^CD20^-^CD19^+^) plasmablasts with RBD IgG titers. Statistics are reported for unexposed and acute samples. (R) Gating strategies for plasmablasts and T cell subtypes. The black dotted line indicates LOD; the green dotted line demarcates marginal responses as determined by unexposed donor responses. Pink dots denote samples where two or more peptide pools were not run due to cell numbers. Acute (Ac) = Red, Convalescent = black, Unexposed (Unexp) = gray. ^∗∗∗^p < 0.001, ^∗∗∗∗^p < 0.0001, NS = not significant.

We examined associations between different branches of adaptive immunity and COVID-19 disease severity. To do this, we first added 11 previously reported convalescent COVID-19 cases (Grifoni et al., 2020a), increasing the cohort size (26 convalescent total, 50 cases total), adding here to the published data on those 11 subjects by performing neutralizing antibody measurements (Figure S4A), *ex vivo* immunophenotyping (Table S3, see below), plasma cytokine measurements (Figures S5A–S5M, see below), and SARS-CoV-2-specific cT_FH_ cell quantitation (Figure S4B). We then examined relationships between peak COVID-19 disease severity and neutralizing antibodies, SARS-CoV-2-specific CD4^+^ T cells, or SARS-CoV-2-specific CD8^+^ T cells across all cases. Presence of neutralizing antibodies was not associated with lessened disease severity (Figure 4E), suggesting that other components of adaptive immunity were important for resolution of SARS-CoV-2 infection. In contrast, SARS-CoV-2-specific CD4^+^ T cells were significantly associated with less severe disease (AIM^+^ CD4^+^ T cells p = 0.0016, two-sided Fisher’s exact test; Figure 4F). SARS-CoV-2-specific CD8^+^ T cells were also associated with less severe disease (AIM^+^ CD8^+^ T cells p = 0.024; Figure 4G). We identified one COVID-19 case that had no detectable neutralizing antibodies and resolved infection without hospitalization (C4844; Figure 1T). This individual had SARS-CoV-2-specific CD4^+^ and CD8^+^ T cells (Figures S4C and S4D), suggestive of an ability of T cell-mediated immunity to control infection.

The antigen-specific SARS-CoV-2 data above suggested that multiple patterns of protective immune responses to SARS-CoV-2 may exist. We therefore took two approaches to examining those potential relationships. The first was a simplified adaptive immunity metric, and the second was a broad-based correlation matrix analysis of all of the antigen-specific measurements. For the first, simplified approach, we stratified COVID-19 cases based on the breadth of the antigen-specific ADIM to SARS-CoV-2: ADIM 0, 1, 2, and 3. Individuals without adaptive immunity by SARS-CoV-2-specific CD4^+^ T cell, SARS-CoV-2-specific CD8^+^ T cell, and neutralizing antibody metrics were categorized as 0. Individuals with only one branch of adaptive immunity measurable against SARS-CoV-2 by antigen-specific assays were categorized as 1, while individuals with two were categorized as 2. Individuals with simultaneous neutralizing antibody, SARS-CoV-2-specific CD4^+^ T cell, and SARS-CoV-2-specific CD8^+^ T cell responses were categorized as ADIM 3. Examples of all four ADIM types were found (Figure S4E). 35% of acute cases and 73% of non-hospitalized convalescent COVID-19 cases fulfilled the ADIM 3 group criteria of successful SARS-CoV-2-specific neutralizing antibody, CD4^+^ T cell, and CD8^+^ T cell responses. Subjects with weak ADIMs were significantly more likely to experience severe COVID-19 disease than subjects with broader ADIMs (p = 0.007, two-sided Fisher’s exact test. (ADIM 0 and 1 versus ADIM 2 and 3; Figure 4H). Thus, broader adaptive immunity was positively associated with protection from severe COVID-19 disease, suggestive of a coordinated ADIM in protective immunity during a SARS-CoV-2 infection.

**Figure S6:**
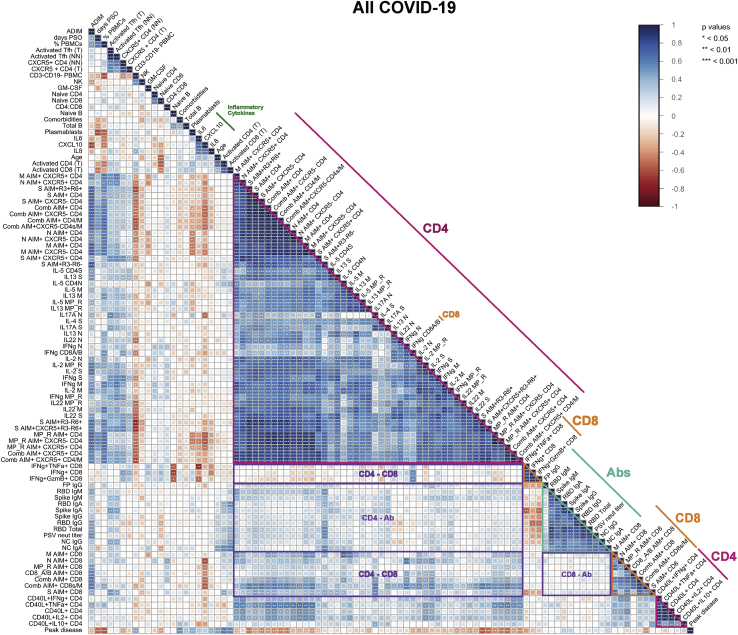
Correlations of the Immune Response with Disease Severity in COVID-19 Donors, Related to Figure 6 Correlogram of all COVID-19 cases. Spearman R values are shown from red (−1.0) to blue (1.0); r values are indicated by color and square size. Peak COVID-19 disease severity (“Peak disease”) is the bottom row. Additional information on feature names are described in the STAR Methods. Blank fields with dots indicate lack of signal. p values are indicated by white asterisks. ^∗^p < 0.05, ^∗∗^p < 0.01, ^∗∗∗^p < 0.001.

**Figure 5:**
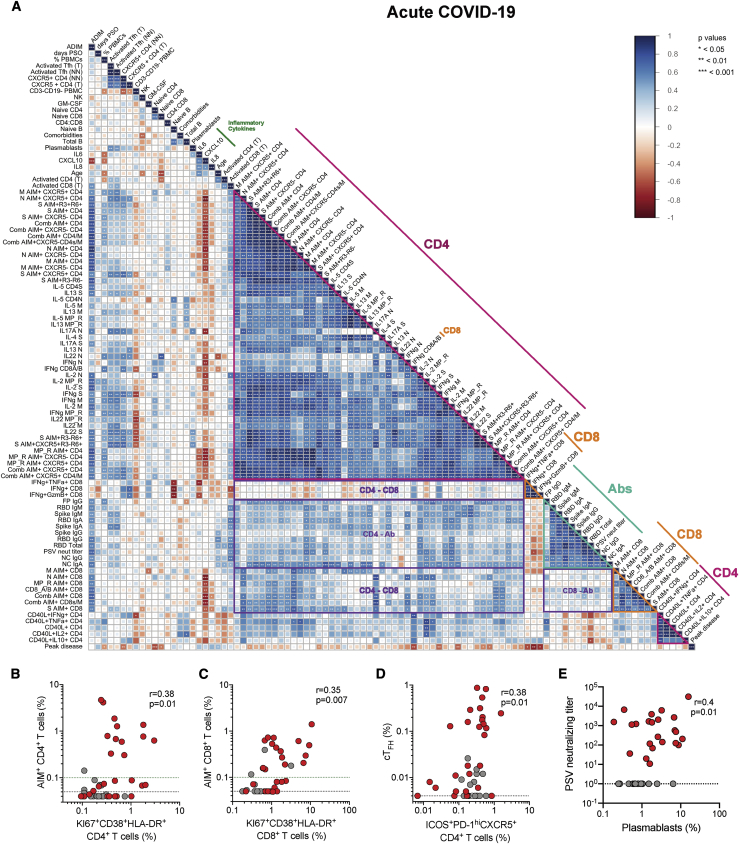
Associations of Adaptive Immune Response Features with COVID-19 Severity (A) Correlogram of acute COVID-19 donors. Spearman rank order correlation values (r) are shown from red (−1.0) to blue (1.0); r values are indicated by color and square size. Blank fields with black dots indicate lack of signal. p values are indicated by white asterisks. The teal triangle denotes SARS-CoV-2 antibody features, magenta triangle denotes SARS-CoV-2-specific CD4^+^ T cells features, and orange triangle denotes SARS-CoV-2-specific CD8^+^ T cell features. Purple rectangles denote coordinated adaptive immune response features. The dark green line denotes select inflammatory cytokines. Peak COVID-19 disease severity (“Peak disease”) is the bottom row. Additional information on feature names are described in the STAR Methods. (B) Correlation of Ki67^+^CD38^+^HLA-DR^+^ CD4^+^ T cells (as percentage of total CD4^+^ T cells) with SARS-CoV-2-specific (combined AIM^+^) CD4^+^ T cells. (C) Correlation of Ki67^+^CD38^+^HLA-DR^+^ CD8^+^ T cells (as percentage of total CD8^+^ T cells) with SARS-CoV-2-specific (combined AIM^+^) CD8^+^ T cells. (D) Correlation of activated (ICOS^+^PD-1^hi^) T_FH_ cells (as percentage of total CD4^+^ T cells) with SARS-CoV-2-specific (combined AIM^+^) T_FH_ (CXCR5^+^CD4^+^) cells. (E) Correlation of SARS-CoV-2 PSV-neutralizing antibody titer and percentage plasmablasts (CD38^hi^CD20^-^ of CD19^+^ B cells). Unexposed controls (n = 15), acute COVID-19 (n = 26) displayed. Statistics reported for (B–E) are reported for unexposed and acute donors. The black dotted line indicates LOD; the green dotted line demarcates marginal responses as determined by unexposed donor responses. ^∗^p < 0.05, ^∗∗^p < 0.01, ^∗∗∗^p < 0.001. See also Figure S5, Table S3, and Data S1.

Next, relationships of immune profiles were examined across 111 parameters, including antigen-specific measurements. The parameters included all antigen-specific CD4^+^ T cell, CD8^+^ T cell, and antibody measurements (Figures 1, 2, 3, 4, S1, S2, S3, and S4), plus general immune cell type measurements, plasma cytokines, age, comorbidities, and COVID-19 clinical disease severity. A broad, 22 parameter immunophenotyping flow cytometry panel of major leukocyte cell types and phenotypic markers was run on all subjects (Table S3). We measured 13 inflammatory proteins in plasma (Figures S5A–S5M). The full data from all acute and convalescent cases were analyzed pairwise by Spearman rank correlations, combined with unsupervised hierarchical clustering, and visualized in correlation heatmap plots (correlograms) of all COVID-19 cases (Figure S6) and acute COVID-19 cases (Figure 5A). Focusing on acute COVID-19 cases, SARS-CoV-2-specific CD4^+^ T cell response specificities (e.g., S, N, M, MP_R) and functionalities (e.g., IFNγ, T_FH_) grouped together in hierarchical clustering, with statistically significant correlations, indicating consistent CD4^+^ T cell response biology to SARS-CoV-2 across most acute COVID-19 patients (Figure 5A). SARS-CoV-2 antibody response specificities and functionalities clustered with significant correlations (Figure 5A). SARS-CoV-2-specific CD8^+^ T cell responses also clustered together with significant correlations (Figure 5A). Many parameters of SARS-CoV-2-specific CD4^+^ T cells and antibodies correlated, as did SARS-CoV-2-specific CD4^+^ and CD8^+^ T cells and several features of SARS-CoV-2-specific CD8^+^ T cells and antibodies (each category of cross-correlations outlined in purple, Figure 5A).

Other studies have used direct *ex vivo* markers of T cell or B cell activation (e.g., KI67) (Mathew et al., 2020), polyclonally stimulated T cells (Lucas et al., 2020; Remy et al., 2020), measurements of common leukocyte types, or inflammatory proteins in blood (Giamarellos-Bourboulis et al., 2020; Laing et al., 2020; Mathew et al., 2020; Meckiff et al., 2020; Ou et al., 2020; Sekine et al., 2020) to obtain powerful information about the status of COVID-19 patients. Such data are much more amenable to large cohort studies and clinical diagnostics; however, direct connections between those parameters and SARS-CoV-2 antigen-specific T and antibody responses remain unclear because of the challenges of obtaining antigen-specific data. Herein, we have focused on measurement of antigen-specific T cells and antibodies against SARS-CoV-2. We therefore cross-compared antigen-specific measurements here with measurements of immunological metrics previously reported in the COVID-19 literature. Surrogate markers for antigen-specific CD4^+^ T cells (CD38^+^HLA-DR^+^KI67^+^), cT_FH_ cells (PD-1^+^ICOS^hi^CXCR5^+^), CD8^+^ T cells (CD38^+^HLA-DR^+^KI67^+^), or B cells (CD20^-^CD38^hi^ plasmablasts) did not group closely with the antigen-specific measurements in hierarchical clustering (Figure 5A). Nevertheless, positive correlations were observed, with activated CD8^+^ T cells correlating reasonably with SARS-CoV-2-specific CD8^+^ T cells (r = 0.55, p = 0.0002; Figures 5C and S5O). SARS-CoV-2-specific CD4^+^ T cells and antibodies exhibited more limited correlation with surrogate markers (SARS-CoV-2-specific CD4^+^ T cells, r = 0.36; Figures 5B and S5P; SARS-CoV-2-specific cT_FH_ cells, r = 0.36, Figure 5D; neutralizing antibodies to plasmablasts, r = 0.4; Figure 5E; RBD IgG to plasmablasts, NS; Figure S5Q). Overall, while surrogate markers provided information, no single cellular parameter served as a strong surrogate for direct measurements of SARS-CoV-2-specific T cells and antibodies.

CXCL10 (IP-10), IL-8, and IL-6 were elevated in acute COVID-19 (p < 0.0001, p < 0.05, p < 0.001; Figures S5A–S5C) and correlated with disease severity (Figure 5A), consistent with large cohort studies of plasma cytokines (Chen et al., 2020; Laing et al., 2020; Mathew et al., 2020; Yang et al., 2020). Lymphocyte percentage was associated with acute disease severity (p = 0.002, CD3^-^CD19^-^ %; Figures 5A and S5N), which has been observed in multiple studies (Laing et al., 2020; Mathew et al., 2020; Zhou et al., 2020a). Notably, correlation plots revealed that while CXCL10 had no correlations with antibody titers, CXCL10 showed strong negative correlations with most SARS-CoV-2-specific CD4^+^ and CD8^+^ T cell features (CXCL10; Figure 5A; e.g., p = 0.0048, r = −0.75, N-specific CD4^+^ T cells (AIM^+^); p = 0.0007, r = −0.84, N-specific CD8^+^ T cells [AIM^+^]), and ADIM score (p < 0.0004, r = −0.69). Thus, CXCL10 is a promising surrogate marker for potentially diagnosing poor SARS-CoV-2-specific CD4^+^ and CD8^+^ T cell responses in patients with acute COVID-19.

### Uncoordinated Adaptive Immunity in Older Individuals, Associated with Scarce Naive CD4^+^ and CD8^+^ T Cells

**Figure 6:**
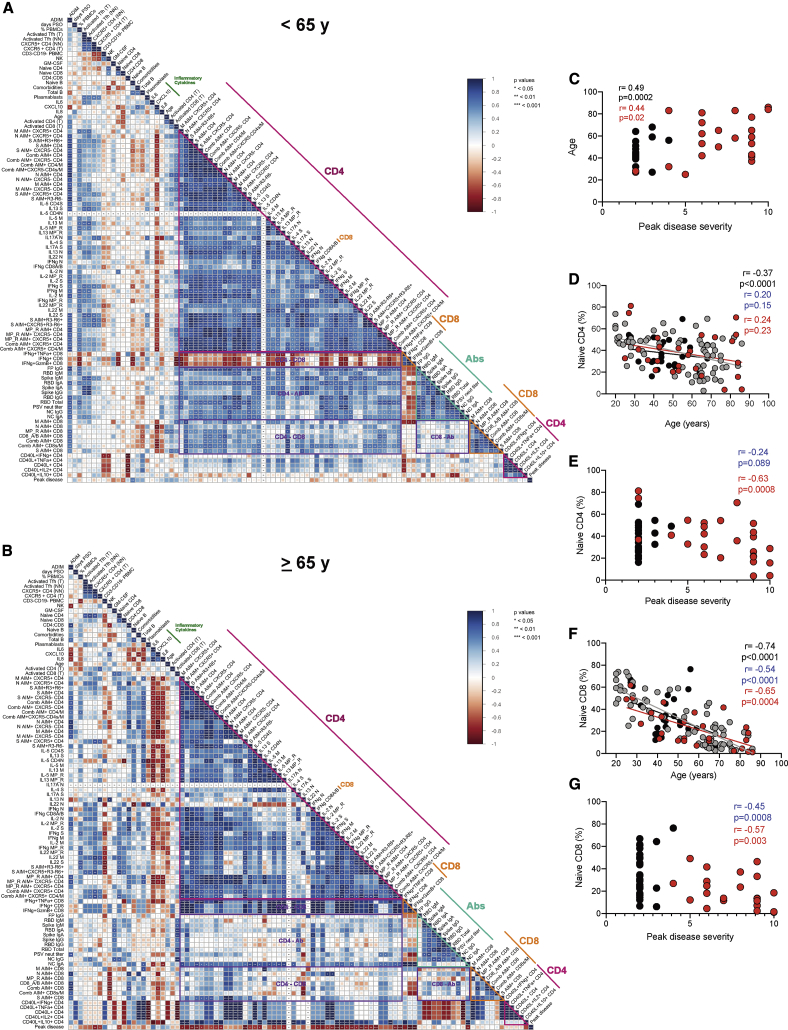
Association of Age and Naive T Cells with COVID-19 Severity (A and B) Correlograms of acute donors < 65 years (A) and ≥ 65 years (B). As in Figure 5, Spearman r correlation values are shown from red (−1.0) to blue (1.0); r values are indicated by color and square size. Blank fields with dots indicate lack of signal. p values are indicated by white asterisks. Also, as in Figure 5, the teal triangle denotes SARS-CoV-2 antibody features, magenta triangle denotes SARS-CoV-2-specific CD4^+^ T cells features, and orange triangle denotes SARS-CoV-2-specific CD8^+^ T cell features. Purple rectangles denote coordinated adaptive immune response features. Peak COVID-19 disease severity (“Peak disease”) is the bottom row. Select inflammatory cytokines are labeled with a dark green line. (C) Correlation of age and peak disease severity. Statistics for full dataset shown are in black; statistics for acute COVID-19 cases are in red. (D) Correlation of naive CD4^+^ T cells (as percentage of total CD4^+^ T cells) with age. Statistics for full dataset are shown in black; statistics for all COVID-19 cases (convalescent and acute) are in blue; statistics for acute COVID-19 cases are in red. (E) Correlation of naive CD4^+^ T cells (as percentage of total CD4^+^ T cells) and peak disease severity. Statistics for all COVID-19 cases (convalescent and acute) are in blue; statistics for acute COVID-19 cases are in red. (F) Correlation of naive CD8^+^ T cells (as percentage of total CD8^+^ T cells) with age. Statistics for full dataset are shown in black; statistics for all COVID-19 cases (convalescent and acute) are in blue; statistics for acute COVID-19 cases are in red. (G) Correlation of naive CD8^+^ T cells (as percentage of total CD8^+^ T cells) and peak disease severity. Statistics for all COVID-19 cases (convalescent and acute) are in blue; statistics for acute COVID-19 cases are in red. Unexposed controls in gray (n = 67), convalescent COVID-19 in black (n = 15), acute COVID-19 in red (n = 26) displayed. ^∗^p < 0.05, ^∗∗^p < 0.01, ^∗∗∗^p < 0.001. See also Figures S6 and S7, Table S3, and Data S1.

**Figure S7:**
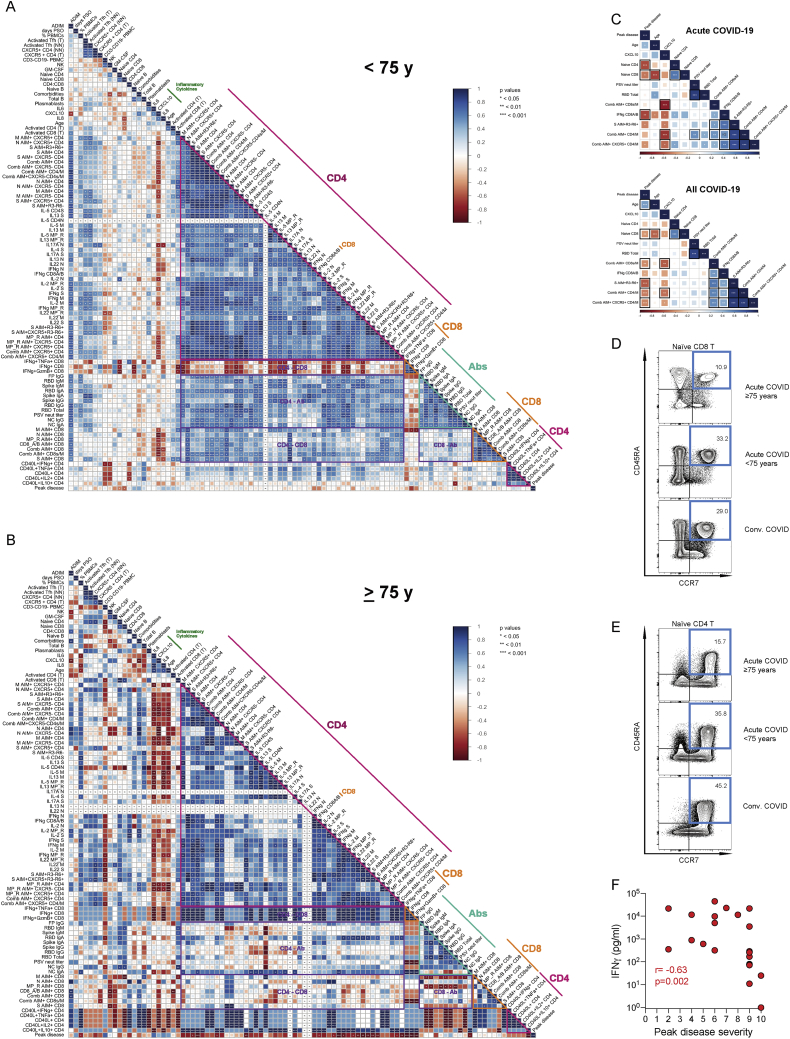
Uncoordinated Adaptive Immunity in the Elderly, Related to Figures 6 and 7 (A-B) Correlograms of all acute COVID-19 cases age < 75 (A) and ≥ 75 (B). Spearman R values are shown from red (−1.0) to blue (1.0). Blank fields with dots indicate lack of signal. (C) Correlogram of curated markers of adaptive immune responses in acute COVID-19 subjects (top) and all COVID-19 subjects (bottom). Spearman r correlation values are shown from red (−1.0) to blue (1.0). ^∗^p < 0.05, ^∗∗^p < 0.001, ^∗∗∗^p < 0.0001. Thick black squares outlining a field indicate adjusted FDR < 0.05. (D-E) Gating strategies for (D) naive CD8^+^ and (E) CD4^+^ T cells for acute and donors < 75 years old and ≥75 years old, and convalescent donors. (F) Secreted IFNg (pg/mL) after SARS-CoV-2 CD8A/B MP stimulation, versus peak COVID-19 disease severity, acute samples (n = 21). Statistics in (F) are reported for acute samples (shown in red). ^∗^p < 0.05, ^∗∗^p < 0.001, ^∗∗∗^p < 0.0001.

Age correlated with COVID-19 disease severity (p = 0.0002, all cases, two-side Spearman rank order correlation test; Figure 6A), which has been widely observed. Notably, correlation plots indicated a relationship between antigen-specific SARS-CoV-2 immune responses and age (“Age”; Figure 5A). We therefore separately assessed immunological interrelationships among older acute COVID-19 cases (Figures 6B, 6C, and S7A–S7C). SARS-CoV-2 ADIMs were quite uncoordinated in patients ≥ 65 years old compared to younger patients (Figure 6B versus 6A). Note the overall reduction in coordination of the CD4^+^ and CD8^+^ T cell responses (purple outlined cluster labeled “CD4 – CD8,” both changes in correlations and statistical significance); dramatic losses in coordination between the CD4^+^ T cell and antibody responses (purple outlined cluster labeled “CD4 – Ab”); and large shifts in correlations between inflammatory cytokines (green line) and CD4^+^ T cell, CD8^+^ T cell, and antibody responses. Changes in ADIM relationships appeared even more altered in patients ≥ 75y old (Figure S7B versus S7A).

Age and COVID-19 disease severity were correlated with multiple immunological characteristics by Spearman correlations analyses (Figures 5, 6, and S7). Intriguingly, correlations were seen between low frequencies of naive CD8^+^ and CD4^+^ T cells, age, and COVID-19 disease severity (Figures 5C–5G, S6, and S7C). To better control for age distributions, naive T cell percentages were examined in 34 additional healthy controls, including 28 individuals ≥ 65y old (n = 65 healthy controls in total; Figures 6D and 6F). Naive CD8^+^ and CD4^+^ T cells (CCR7^+^CD45RA^+^) strongly correlated with age and were less than 10% of CD8^+^ T cells in some individuals (CD8 r = −0.74. p < 0.0001; Figure 6F; CD4 r = −0.37, p < 0.0001; Figure 6D). The relationship between naive T cell % and age was indistinguishable between healthy controls and acute COVID-19 cases, or convalescent COVID-19 cases (ANCOVA multivariate analysis, visualized by overlapping linear regressions; Figures 6D and 6F), with clear overlap in the naive T cell frequencies of cases and controls. We then examined the relationship between naive T cells and COVID-19 severity. Naive CD8^+^ T cell percentage was associated with peak COVID-19 disease severity among acute patients (r = −0.57, p = 0.003; Figure 6G), and that relationship was maintained when considering all COVID-19 cases (acute and convalescent. r = −0.45, p = 0.0008; Figure 6G), indicating that low naive CD8^+^ T cell percentage was not simply an effect of acute COVID-19. Low naive CD4^+^ T cell percentage correlated with COVID-19 disease severity among acute patients (p = 0.0008), but the relationship was lost when considering all COVID-19 cases (acute and convalescent. p = 0.09; Figure 6E), suggesting that the low naive CD4^+^ T cell percentage may primarily be a consequence of acute disease, unlike the low naive CD8^+^ T cell percentage. Thus, scarce naive CD8^+^ T cells were associated with risk of severe COVID-19. New antigen-specific responses depend on the pool of naive lymphocytes. A small starting pool of naive CD8^+^ and/or CD4^+^ T cells may limit the likelihood of priming a fast or large virus-specific T cell response due to the reduced starting material.

### Strongest Associations between COVID-19 Severity and Antigen-Specific Immune Responses

**Figure 7:**
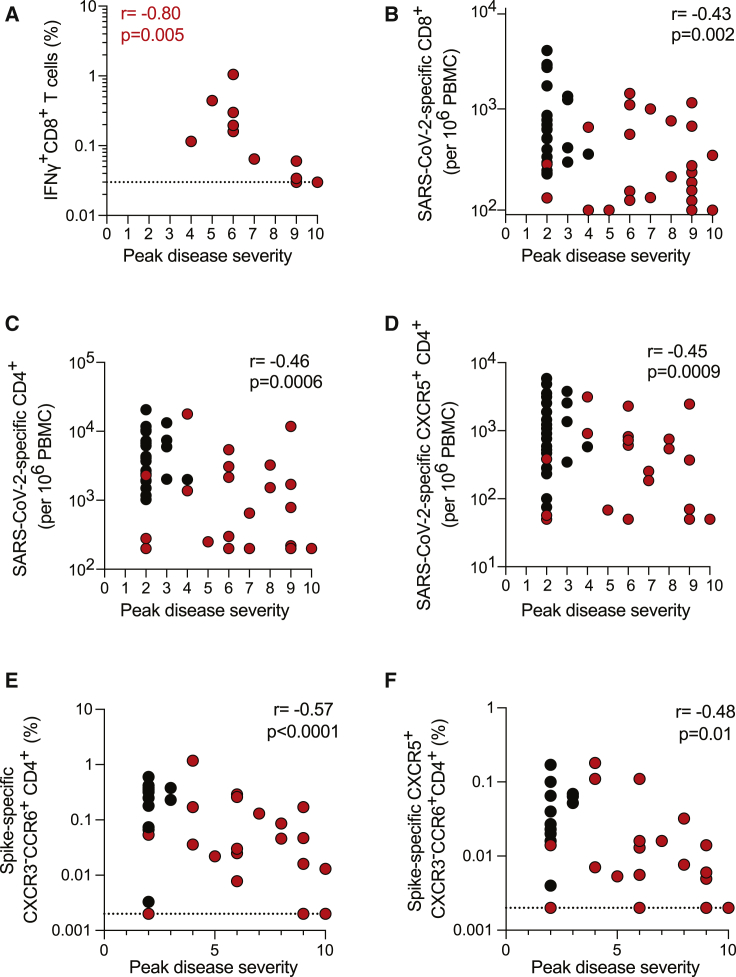
Associations of COVID-19-Specific CD4 and CD8 T Cell Responses and Disease Severity (A) Frequency of IFNγ^+^ CD8^+^ T cells in response to CD8A/B MP ICS, versus peak COVID-19 disease severity, acute donors (n = 11 samples). Dotted line indicates LOD. (B–D) Associations between peak COVID-19 disease severity and number per million PBMC of (B) AIM^+^ CD8^+^ T cells (CD69^+^4-1BB^+^CD8^+^), (C) AIM^+^ CD4^+^ T cells (OX40^+^surfaceCD40L^+^), or (D) AIM^+^ cT_FH_ cells (OX40^+^CD40L^+^CXCR5^+^CD4^+^) across all SARS-CoV-2 peptide-specific MPs, acute samples (n = 26), and convalescent samples (n = 15). (E and F) Frequency of (E) CXCR3^-^CCR6^+^ S-specific (AIM^+^) CD4^+^ T cells and (F) CXCR5^+^CXCR3^-^CCR6^+^ S-specific (AIM^+^) cT_FH_ CD4^+^ T cells versus peak disease severity, acute samples (n = 26), and convalescent samples (n = 15). Dotted line denotes LOD. Statistics for acute COVID-19 cases are in red; statistics for all COVID-19 cases (convalescent and acute) are in black. See also Figure S7 and Data S1.

Given the overall analyses above, we examined which antigen-specific immune responses exhibited the strongest association with COVID-19 disease severity (“peak disease,” bottom row Figure 5 acute; bottom row Figure S6 all COVID-19). The strongest associations with low disease severity among acute cases were IFNγ-producing CD8^+^ T cells (Figure 7A, IFNγ ICS r = −0.80, p = 0.005; Figure S7F, IFNγ cytokine secretion r = −0.63, p = 0.002). The strongest associations with low disease severity among total cases included the total SARS-CoV-2-specific CD8^+^ T cells (per 10^6^ PBMCs, r = −0.43, p = 0.002; Figure 7B), with even stronger association for SARS-CoV-2-specific CD4^+^ T cells (per 10^6^ PBMCs, r = −0.46, p = 0.0006; Figure 7C) as well as the SARS-CoV-2-specific cT_FH_ (per 10^6^ PBMCs, r = −0.45, p = 0.0009; Figure 7D). Notably, both S-specific CXCR3^-^CCR6^+^ CD4^+^ T cells and S-specific CXCR3^-^CCR6^+^ cT_FH_ were associated with low disease severity (r = −0.57, p = 0.0001, Figure 7E; r = −0.48, p = 0.01, Figure 7F). IL-17a expression was generally not observed (Figure S2F) and was not associated with disease severity (Figure S6). IL-22 expression was observed but was also minimally associated with lower disease severity (Figure 6), suggesting that the CCR6 expression by these CD4^+^ T cells may primarily reflect lung-homing characteristics. Statistically significant associations were generally not observed between SARS-CoV-2 antibodies and disease severity (Figures 5 and S6). Overall, associations were found between strong SARS-CoV-2-specific T cell responses and low COVID-19 disease severity.

## Discussion

Understanding of immunity to COVID-19 is growing but remains limited, and furthering our understanding depends on measuring all three branches of adaptive immunity in a SARS-CoV-2 antigen-specific manner in acute COVID-19 patients. While this study was exploratory in nature, the antigen-specific antibody and T cell data here suggest the following: (1) ADIMs limit COVID-19 disease severity; (2) coordinated responses by all three branches of adaptive immunity were better than partial responses, with prominent roles for SARS-CoV-2-specific CD4^+^ T cells associated with less COVID-19 disease severity; (3) CXCL10 may be a plasma marker in acute COVID-19 of impaired T cell responses; and (4) aging and scarcity of naive T cells may be linked risk factors for failure to generate a coordinated ADIM, resulting in increased susceptibility to severe COVID-19. These findings have implications both for understanding COVID-19 immunity and pathology, as well as COVID-19 vaccine designs. Future studies will be required to test these relationships rigorously.

Neutralizing antibody titers were not predictive of reduced disease severity in this cohort as an individual parameter. Instead, broad and coordinated ADIMs were associated with lesser COVID-19 disease severity, while absent or minimal adaptive immunity was associated with more severe COVID-19 disease. SARS-CoV-2-specific CD4^+^ T cells were associated with protective immune responses in this cohort. Significant redundancy or compensation may exist between the protective actions of neutralizing antibodies, SARS-CoV-2-specific CD4^+^ T cells, and SARS-CoV-2-specific CD8^+^ T cells. Neutralizing antibody titers were associated with protection against SARS-CoV-2 in non-human primate infection and rechallenge studies (Chandrashekar et al., 2020; Gao et al., 2020), as well as in three candidate COVID-19 vaccine studies in non-human primates (Gao et al., 2020; Wang et al., 2020c; Yu et al., 2020). However, it is easier for antibodies to provide protective immunity when present before exposure to the pathogen (prophylactic). In many viral infections, CD4^+^ and CD8^+^ T cells are key for control and clearance of an acute infection. Additionally, T_FH_ cells are required for most IgG responses and high-quality neutralizing antibodies. The different arms of adaptive immunity can compensate for each other in protective immunity in some contexts (Amanna et al., 2006; Plotkin et al., 2018). For example, neutralizing antibodies, CD4^+^ T cells, and CD8^+^ T cells have each individually been shown to have protective roles against poxvirus infections in mice, and depletion of any one of the three components of the adaptive immune system can still result in protection from vaccinia virus infection (Amanna et al., 2006; Salek-Ardakani et al., 2008). One study stratified subgroups of severe COVID-19 cases based on inferred phenotypic markers of adaptive immunity (e.g., plasmablasts or HLA-DR^+^ T cells) and concluded that one subgroup was represented by patients with low to undetectable activation of T and B cells (Mathew et al., 2020), though antigen-specific T cells and neutralizing antibodies were not directly measured. Those data are consistent with our findings. Together, the available data suggest that coordinated adaptive immunity by all three branches of adaptive immunity is likely to be beneficial in minimizing COVID-19 severity, as is seen in protection against other infectious diseases.

Low frequencies of naive T cells were immunological risk factors associated with severe COVID-19 disease in this cohort. A repertoire of fewer naive T cells in older individuals (Briceño et al., 2016; Qi et al., 2014) may be exacerbated as a risk factor specifically for severe COVID-19, because early innate immune evasion by SARS-CoV-2 may limit T cell priming (Blanco-Melo et al., 2020; Vabret et al., 2020), and there is the possibility of fewer professional antigen presenting cells in the lungs with advanced age, as has been seen in small animal models of SARS (Chen et al., 2010; Zhao et al., 2011). The detrimental effects of fewer naive T cells may also be amplified by the well-characterized total lymphopenia (Wang et al., 2020a) and general T cell cytopenia observed in severe COVID-19 (Wang et al., 2020b). This may be further exacerbated by the uncoordinated SARS-CoV-2-specific immune response to COVID-19 observed here in older patients.

The adaptive immune system has the capacity to cause immunopathogenesis. We found little evidence to support hypotheses of pathogenic adaptive immune cells being causally involved in COVID-19 pathogenesis. Hospitalized COVID-19 patients did not have T_H_2 or T_H_17 cytokine skewed CD4^+^ T cell responses, consistent with most other reports (Meckiff et al., 2020; Sekine et al., 2020; Weiskopf et al., 2020); and the CD8^+^ T cell response cytokine profile was similar between hospitalized and non-hospitalized cases. While we do not exclude the possibility of some functional T cell defects, the data here largely supported a model wherein a slow or uncoordinated (partial) ADIM was associated with severe disease. Conversely, strong SARS-CoV-2-specific CD4^+^ or CD8^+^ T cell responses were associated with low disease severity. Our adaptive immunity findings are consistent with findings that dysregulated innate immunity may be central to COVID-19 associated immunopathogenesis.

COVID-19 vaccine development is a topic of major importance. A vaccine does not have to directly mimic protective immunity observed in natural infection but should be informed by protective immunity observed in natural infection. Resolving an ongoing infection is more challenging than prophylaxis. The data presented here suggest that neutralizing antibodies play a role in resolving acute COVID-19, but statistical associations found less of a role for antibodies than SARS-CoV-2-specific CD4^+^ or CD8^+^ T cells. These results suggest that vaccine approaches that elicit antiviral SARS-CoV-2-specific CD4^+^ and CD8^+^ T cells in coordination with neutralizing antibodies will generate protective immunity that most closely analogous to the coordinated adaptive antiviral immune response seen in most cases of COVID-19 following natural SARS-CoV-2 infection.

## Limitations of Study

Caveats of this study include the sample size and sampling of blood. While antigen-specific immunity data are reported for a total of 50 COVID-19 cases here, robust testing will require larger cohorts. While comorbid medical conditions and other factors play a role in COVID-19 disease severity, this study was not powered to distinguish such factors. Independent test sets will also be important in future studies. While blood sampling is a necessity for the vast majority of human immunology studies, it is established that certain cell types of ADIMs can be restricted to organs, such as the lungs, and not be detectable in the circulation (Masopust and Soerens, 2019). However, SARS-CoV-2-specific CD4^+^ T cells, SARS-CoV-2-specific CD8^+^ T cells, and neutralizing antibodies were detected in the blood of most COVID-19 cases in this study, indicating the experimental approach was informative and the conclusions are reasonable. Additionally, while immunological data from lungs of COVID-19 cases are limited, the data on total abundance of CD8^+^ T cells in COVID-19 lungs (Liao et al., 2020) are consistent with our conclusion that weaker SARS-CoV-2-specific T cell responses are associated with worse disease. The data reported here do not assess the role of pre-existing cross-reactive T cells in response to SARS-CoV-2 (Braun et al., 2020; Grifoni et al., 2020a; Meckiff et al., 2020; Weiskopf et al., 2020), as the antigen-specific tools used in this study did not distinguish between T cell responses post-infection that came from cross-reactive or *de novo* epitope specificities.

In sum, we quantified and phenotyped SARS-CoV-2-specific CD4^+^ T cells, SARS-CoV-2-specific CD8^+^ T cells, and antibody responses in both acute and convalescent COVID-19 cases. Using multiple experimental approaches, we connected key relationships between SARS-CoV-2 antigen-specific T cell immunological features, COVID-19 disease severity, aging, and other features. These data are valuable antigen-specific foundations for understanding clinical aspects of COVID-19 and COVID-19 vaccine efforts.

## STAR★Methods

### Key Resources Table

**Table:** 

REAGENT or RESOURCE	SOURCE	IDENTIFIER
**Antibodies**		

Ki-67-FITC human	BD Biosciences	BD Biosciences Cat# 556026; RRID:AB_396302, clone B56
CD4 Monoclonal Antibody (SK3 (SK-3)), PerCP-eFluor 710, eBioscience	Thermo Fisher Scientific	Thermo Fisher Scientific Cat# 46-0047-42; RRID:AB_1834401, clone SK3
Alexa Fluor 647 anti-human/mouse Granzyme B antibody	BioLegend	BioLegend Cat# 515406; RRID:AB_2566333, clone GB11
Alexa Fluor 700 anti-human IgM antibody	BioLegend	BioLegend Cat# 314538; RRID:AB_2566615, clone MHM-88
APC/Cyanine7 anti-human CD27 antibody	BioLegend	BioLegend Cat# 302816; RRID:AB_571977, clone O323
Mouse Anti-IgD Monoclonal Antibody, Phycoerythrin Conjugated, Clone IA6-2	BD Biosciences	BD Biosciences Cat# 555779; RRID:AB_396114, clone IA6-2
PE/Dazzle 594 anti-human CD56 (NCAM) antibody	BioLegend	BioLegend Cat# 318348; RRID:AB_2563564, clone HCD56
Mouse Anti-HLA-DR, DP, DQ Monoclonal Antibody, Unconjugated, Clone TU39	BD Biosciences	BD Biosciences Cat# 555557; RRID:AB_395939, clone TU39
PE/Cy5 anti-human CD19 antibody	BioLegend	BioLegend Cat# 302210; RRID:AB_314240, clone HIB19
CD38 Monoclonal Antibody (HIT2), PE-Cyanine7, eBioscience	Thermo Fisher Scientific	Thermo Fisher Scientific Cat# 25-0389-42; RRID:AB_1724057, clone HIT2
Brilliant Violet 421 anti-human CD185 (CXCR5) antibody	BioLegend	BioLegend Cat# 356920; RRID:AB_2562303, clone J252D4
Brilliant Violet 510 anti-human CD14 antibody	BioLegend	BioLegend Cat# 367123; RRID:AB_2716228, clone 63D3
Brilliant Violet 570 anti-human CD45RA antibody	BioLegend	BioLegend Cat# 304132; RRID:AB_2563813, clone HI100
Brilliant Violet 605 anti-human CD183 (CXCR3) antibody	BioLegend	BioLegend Cat# 353728; RRID:AB_2563157, clone G02H57
Brilliant Violet 650 anti-human CD20 antibody	BioLegend	BioLegend Cat# 302336; RRID:AB_2563806, clone 2H7
Brilliant Violet 711 anti-human CD197 (CCR7) antibody	BioLegend	BioLegend Cat# 353228; RRID:AB_2563865, clone G043H7
Brilliant Violet 785 anti-human CD279 (PD-1) antibody	BioLegend	BioLegend Cat# 329930; RRID:AB_2563443, clone EH12.2H7
BUV396 CD3 antibody human	BD Biosciences	BD Biosciences Cat# 563546; RRID:AB_2744387, clone UCHT1
BD Horizon BUV496 Mouse Anti-Human CD196 (CCR6) antibody	BD Biosciences	BD Biosciences Cat# 612948; RRID:AB_2833076, clone 11A9
BUV737 Mouse Anti-Human CD16 antibody	BD Biosciences	BD Biosciences Cat# 612786; RRID:AB_2833077, clone 3G8
BD Horizon BUV805 Mouse Anti-Human CD8 antibody	BD Biosciences	BD Biosciences Cat# 612889; RRID:AB_2833078, clone SK1
LIVE/DEAD Fixable Blue Dead Cell Stain Kit, for UV excitation	Thermo Fisher Scientific	Thermo Fisher Scientific Cat# L34962
FITC anti-human CD69 antibody	BioLegend	BioLegend Cat# 310904; RRID:AB_314839, clone FN50
APC anti-human CD134 (OX40) antibody	BioLegend	BioLegend Cat# 350008;; RRID:AB_10719958, Clone Ber-ACT35 (ACT35)
PE anti-human CD274 (B7-H1, PD-L1) antibody	BioLegend	BioLegend Cat# 329706; RRID:AB_940368, Clone 29E.2A3
PE/Dazzle 594 anti-human CD154 antibody	BioLegend	BioLegend Cat# 310840; RRID:AB_2566245, Clone 24-31
Brilliant Violet 510 anti-human CD25 antibody	BioLegend	BioLegend Cat# 302640; RRID:AB_2629672, Clone BC96
BUV563 Mouse Anti-Human CD278	BD OptiBuild	BD Biosciences Cat# 741421, clone DX29
CD14 Monoclonal Antibody (61D3), APC-eFluor 780, eBioscience	Thermo Fisher Scientific	Thermo Fisher Scientific Cat# 47-0149-42; RRID:AB_1834358, Clone 61D3
CD16 Monoclonal Antibody (eBioCB16 (CB16)), APC-eFluor 780, eBioscience	Thermo Fisher Scientific	Thermo Fisher Scientific Cat# 47-0168-42; RRID:AB_11220086, Clone eBioCB16 (CB16)
CD20 Monoclonal Antibody (2H7), APC-eFluor 780, eBioscience	Thermo Fisher Scientific	Thermo Fisher Scientific Cat# 47-0209-42; RRID:AB_1272038, Clone 2H7
IL-13 Monoclonal Antibody (85BRD), FITC, eBioscience	Thermo Fisher Scientific	Thermo Fisher Scientific Cat# 11-7136-42; RRID:AB_2572515, Clone 85BRD
Alexa Fluor 700 anti-human IL-2 antibody	BioLegend	BioLegend Cat# 500320; RRID:AB_528929, Clone MQ1-17H12
PE/Dazzle 594 anti-human IL-10 antibody	BioLegend	BioLegend Cat# 506812; RRID:AB_2632783, Clone JES3-19F1
TNF alpha Monoclonal Antibody (MAb11), PE-Cyanine7, eBioscience	Thermo Fisher Scientific	Thermo Fisher Scientific Cat# 25-7349-82; RRID:AB_469686, Clone MAb11
Brilliant Violet 785 anti-human IL-17A antibody	BioLegend	BioLegend Cat# 512338; RRID:AB_2566765, Clone BL168
BUV737 Mouse Anti-Human IFN-g Clone 4S.B3	BD Biosciences	BD Biosciences Cat# 612845, Clone 4S.B3
Anti-Human IgG (gamma-chain specific)-Peroxidase antibody produced in goat	Sigma-Aldrich	Sigma-Aldrich Cat# A6029; RRID:AB_258272
Goat Anti-Human IgM Polyclonal Antibody, Horseradish Peroxidase Conjugated	Sigma-Aldrich	Sigma-Aldrich Cat# A6907; RRID:AB_258318
Human IgA Fd PAN (A1/2)	Hybridoma Reagent Laboratory	Hybridoma Reagent Laboratory HP6123-HRP
Mouse IgG1 anti-Human IgA Fd PAN (A1/2)	Hybridoma Reagent Laboratory	Hybridoma Reagent Laboratory Cat# HP6123-HRP
Polyclonal human sera	This study	
Human polyclonal sera	This study	

**Bacterial and Virus Strains**		

SARS-CoV-2-nanoLuc virus (WA1 strain) in which ORF7 was replaced by nanoluciferase gene (nanoLuc) was generated by reverse genetics	Hou et al., 2020	GenBank: MT461671.1
Pseudotyped DG-luciferase (G^∗^DG-luciferase) rVSV	Kerafast	Kerafast Cat# EH1020-PM
rVSV-SARS-CoV-2	This study	

**Biological Samples**		

Healthy unexposed donor blood samples	UC San Diego Health	
Convalescent COVID-19 donor blood samples	LJI Clinical Core	https://www.iedb.org/
Acute and convalescent COVID-19 donor blood samples	UC San Diego Health	

**Chemicals, Peptides, and Recombinant Proteins**		

Synthetic peptides	Synthetic biolmolecules (aka A&A)	http://syntheticbiomolecules.com
Electron Microscopy Sciences 16% Paraformaldehyde Aqueous Solution, EM Grade, Ampoule 10 ML	Electron Microscopy Sciences	Electron Microscopy Sciences Cat# 15710
Thermo Scientific Hoechst 33342 Solution (20 mM)	Thermo Scientific	Thermo Scientific Cat# 62249
Mirus Bio *Trans*IT-LT1 Transfection Reagent	Mirus Bio	Mirus Bio Cat# MIR 2304
recombinant SARS-CoV-2 Spike protein	This study	
recombinant SARS-CoV-2 Spike protein receptor binding domain (RBD) protein	This study	
Quick-RNA Viral Kit	Zymo Research	Zymo Research Cat # R1035, Lot # ZRC205587
TaqMan Fast Virus 1-Step Master Mix	Applied Biosystems	Applied BiosystemsCat # 4444434, Lot # 00900184

**Critical Commercial Assays**		

*CoronaCheck*COVID-19 Rapid Antibody Test Kit	20/20 BioResponse	https://coronachecktest.com/

**Experimental Models: Cell Lines**		

Vero	ATCC	ATCC Cat# CCL-81, RRID:CVCL_0059
HEK293T	ATCC	ATCC Cat# CRL-3216, RRID:CVCL_0063

**Oligonucleotides**		

nCOV_N1 Reverse Primer Aliquot	Integrated DNA Technologies	IDT Cat# 10006831, Lot #0000515800
nCOV_N1 Probe Aliquot	Integrated DNA Technologies	IDT Cat# 10006823, Lot #0000515803
2019-nCoV_N Positive Control	Integrated DNA Technologies	IDT Cat# 10006625, Lot #0000509951
nCOV_N1 Forward Primer Aliquot	Integrated DNA Technologies	IDT Cat# 10006830, Lot #0000515799

**Recombinant DNA**		

phCMV3-SARS-CoV-2	This study; Spike cloned from synthetic, codon optimized DNA	
Empty vector: phCMV3	Genlantis	Gelantis Cat# P003300
pCAGGS-VSV-G	Kerfast	Kerfast Cat# EH1017

**Software and Algorithms**		

LEGENDplex v8.0	BioLegend	https://www.biolegend.com/
GraphPad Prism 8.4	GraphPad	https://www.graphpad.com/
FlowJo 10	FlowJo	https://www.flowjo.com/
IEDB	Grifoni et al., 2020a	https://www.iedb.org
corrplot package (v0.84) running under R (v3.6.1) in Rstudio(1.1.456)	Wei and Sikmo, 2017	https://github.com/taiyun/corrplot

**Other**		

CellInsight CX5 High-Content Screening (HCS) Platform	Thermo Scientific	Thermo Scientific Cat# CX51110

### Resource Availability

#### Lead Contact

Further information and requests for resources and reagents should be directed to and will be fulfilled by the Lead Contact, Shane Crotty (shane@lji.org).

#### Materials Availability

Aliquots of the synthesized peptides or plasmids used in this study will be made available upon request. There are restrictions to the availability of these reagents due to cost and limited quantities.

#### Data and Software Availability

Original data have been deposited to Mendeley Data: http://doi.org/10.17632/n66n5pj4f6.2.

### Experimental Model and Subject Details

#### Human Subjects

##### Healthy Unexposed Donors

Blood samples from healthy adult donors were obtained via phlebotomy under a protocol approved by the Institutional Review Board of the University of California, San Diego (UCSD; 180752) and accepted by the Institutional Review Board of the La Jolla Institute (LJI) under a reliance agreement. These blood samples were collected for studies unrelated to COVID-19 between September 2018 and October 2019. At the time of enrollment in the initial studies, all individual donors provided informed consent that their samples could be used for future studies, including this study.

These samples were considered to be from unexposed controls given that SARS-CoV-2 emerged as a novel pathogen in late 2019 (November to December), and these samples were largely collected in 2018 or the first half of 2019. Blood from the last unexposed donor was collected in October 2019, well before the identification of COVID-19 community spread in San Diego. This donor had no known history of travel to Wuhan, China or another area with potential COVID-19 transmission prior to donation, and did not have symptoms consistent with COVID-19 at the time of donation. These donors were considered healthy in that they had no known history of any significant systemic illnesses, including but not limited to anemia, diabetes, kidney or liver disease, cardiovascular disease, malignancy, or coagulopathy. The presence of any significant systemic illness was considered an exclusion criterion. Inclusion criteria included age 18 years or older at the time of enrollment, males or non-pregnant, non-nursing females, without any of the aforementioned health conditions or other significant health conditions, weighing at least 110 pounds, not on aspirin or anticoagulants for at least five days prior, and with normal vital signs and self-reported good health at the time of the blood draw. An overview of the characteristics of these unexposed donors is provided in **Table S1**.

An additional 50 healthy donors were added to the study to balance the representation of healthy, unexposed individuals in the study, specifically to increase the number of healthy older age individuals. This increased the total number of healthy, unexposed donor samples to 65. In addition to blood samples from prior LJI cohorts who had provided informed consent that their samples could be used for future studies (including this study), blood samples were obtained by LJI from the San Diego Blood Bank. Individuals who donated at the San Diego Blood Bank were considered healthy and safe to donate at the time of donation per the San Diego Blood Bank’s blood donation policies. An IRB approved protocol was not needed to obtain these samples at the time of collection, as this was not deemed to be human subjects’ research.

##### Convalescent COVID-19 Donors

Convalescent donors were either referred to the study by a health care provider or self-referred. Blood samples from convalescent donors were obtained via phlebotomy under protocols approved by the Institutional Review Boards of UCSD (200236X) and LJI (VD-214). All human subjects were assessed for capacity using a standardized and approved assessment. Subjects deemed to have capacity voluntarily gave informed consent prior to being enrolled in the study. Individuals did not receive compensation for their participation in the study.

Study inclusion criteria included subjects with a clinical and/or laboratory diagnosis of COVID-19 over the age of 18 years, regardless of disease severity, race, ethnicity, gender, pregnancy or nursing status, or the presence of other medical conditions, who were willing and able to provide informed consent. Study exclusion criteria included lack of willingness or ability to provide informed consent, or lack of an appropriate legal guardian or representative to provide informed consent. Subjects could be excluded if blood donation was deemed to be medically unsafe or otherwise not in the best medical interest of the subject.

Blood from convalescent donors was obtained via phlebotomy at a UC San Diego Health clinic. Whole blood was collected in acid citrate dextrose (ACD) tubes and stored at room temperature briefly prior to processing for PBMC and plasma isolation. Whole blood was separately collected in serum separator tubes (SST) and stored briefly at room temperature prior to serum isolation. The maximum blood volume collected (for any purposes) within any 8-week period was set at 550 mL per the IRB-approved protocols. Samples were de-identified prior to analysis. Other efforts to maintain the confidentiality of participants included referring to specimens and other records via an assigned, coded identification number.

Prior to enrollment in the study, donors were asked to provide proof of positive PCR-based testing for SARS-CoV-2 (if available), and screened for clinical history and/or epidemiological risk factors consistent with the World Health Organization (WHO) or Centers for Disease Control and Prevention (CDC) case definitions of COVID-19 or Persons Under Investigation (PUI) (https://www.who.int/emergencies/diseases/novel-coronavirus-2019/technical-guidance-publications, https://www.cdc.gov/coronavirus/2019-nCoV/hcp/clinical-criteria.html). Per CDC and WHO guidance, clinical features consistent with COVID-19 included subjective or measured fever, signs or symptoms of lower respiratory tract illness (e.g., cough or dyspnea). Epidemiologic risk factors included close contact with a laboratory-confirmed case of SARS-CoV-2 within 14 days of symptom onset or a history of travel to an area with a rate of COVID-19 cases within 14 days of symptom onset.

Convalescent donors were screened for symptoms prior to scheduling blood draws, and had to be symptom-free and approximately 3 weeks out from symptom onset at the time of the initial blood draw. Following enrollment, whole blood from most convalescent donors was run on a colloidal-gold immunochromatographic assay to evaluate for prior exposure to SARS-CoV-2. This assay detects IgM or IgG antibodies directed against recombinant SARS-CoV-2 antigen labeled with a colloidal gold tracer (20/20 BioResponse CoronaCheck). All of the convalescent donors tested positive for IgM or IgG to SARS-CoV-2 by this assay (**Table S1**).

An overview of the characteristics of the 15 new convalescent donors is provided in Table S1. Complete demographics data was not consistently collected at the time that these convalescent donors were enrolled, and race and ethnicity data are not available for these individuals. However, these donors were all recruited and enrolled in San Diego County. Per the 2019 US Census Bureau data for San Diego County (2019), approximately 75% of San Diego County residents identify as White alone, 6% as Black or African American alone, just over 1% as American Indian or Alaskan Native alone, 13% as Asian alone, just under 1% as Native Hawaiian or other Pacific Islander alone, and 5% as biracial or multiracial. Regarding ethnicity, 34% identify as Hispanic or Latino. The majority (87%) of convalescent donors had a known sick contact with COVID-19 or suspected exposure to SARS-CoV-2 (**Table S1**). The most common symptoms reported were cough, fatigue, fever, dyspnea, and anosmia (**Table S1**). Peak disease severity and disease severity at the time of blood collection was classified as described in the acute disease COVID-19 donor section below and **Table S2**. Seventy-three percent of donors experienced mild illness (**Table S1**). Donors were asked to self-report any known medical illnesses. Of note, 53% of these individuals had no known underlying medical illnesses (**Table S1**). The convalescent donors from the prior publication are described in that publication (Grifoni et al., 2020a)

##### Acute disease COVID-19 Donors

The Institutional Review Boards of the University of California, San Diego (UCSD; 200236X) and La Jolla Institute (LJI; VD-214) approved blood draw protocols for donors with acute COVID-19. All human subjects were assessed for capacity using a standardized and approved assessment. Subjects deemed to have capacity voluntarily gave informed consent prior to being enrolled in the study. When subjects were deemed to lack capacity, they were enrolled only if an appropriate surrogate was identified and gave informed consent. Individuals or their surrogates did not receive compensation for their participation in the study.

Study inclusion criteria included subjects with a diagnosis of COVID-19 and positive PCR-based testing for SARS-CoV-2 and ongoing symptoms and/or clinical findings consistent with acute COVID-19, who were over the age of 18 years, regardless of disease severity, race, ethnicity, gender, pregnancy or nursing status, who were willing and able to provide informed consent or with a legal guardian or representative willing and able to provide informed consent when the participant could not personally do so. Study exclusion criteria included lack of willingness or ability to provide informed consent or lack of an appropriate legal guardian or representative to provide informed consent, or clinically significant anemia or another medical contraindication to blood donation.

Other than for 2 donors who were never hospitalized, blood was obtained from donors with acute, symptomatic COVID-19 at various stages of illness while hospitalized within the UC San Diego Health system (UCSD) at either the Hillcrest or La Jolla campus (3 hospitals in total including the Jacobs Medical Center and Sulpizio Cardiovascular Center in La Jolla). UCSD provides indigent care, serves as a major tertiary and quaternary referral center for San Diego, Riverside, and Imperial counties, and offers specialty care (including comprehensive surgical, HIV, cardiovascular, transplant and oncologic care) not available at other hospitals in the region. Consequently, UCSD provides care for a more diverse and complex patient population than is reflected by the San Diego county population alone. All subjects were assessed for positive SARS-CoV-2 PCR-based testing, clinical disease and/or chest imaging consistent with COVID-19 prior to enrollment. The majority of these donors were identified as having COVID-19 based on a positive PCR-based test for SARS-CoV-2 (GenMark ePlex SARS-CoV-2 Test for SARS-CoV-2, Roche cobas SARS-CoV-2, Abbott m2000 COVID-19, GenMark ePlex SARS-CoV-2, or Luminex ARIES SARS-CoV-2 assay) or a rapid point of care molecular test for SARS-CoV-2 (Abbott ID NOW COVID-19) performed at the UCSD Center for Advanced Laboratory Medicine or performed onsite at the (UCSD Hillcrest or La Jolla) hospital clinical laboratory, respectively. Donors were also included if they had positive testing for SARS-CoV-2 at an outside laboratory based on clinical documentation, self-report, or confirmation of positive testing from an outside laboratory.

Whole blood from all hospitalized donors was collected in EDTA tubes and stored at room temperature briefly prior to processing for PBMC and plasma isolation. Blood from the two non-hospitalized donors was collected in ADC tubes. Whole blood was also collected in additional serum separator tubes (SST) for serum isolation. The maximum blood volume collected (for any purposes) within any 8-week period was set at 550 mL per the IRB-approved protocols. Samples were de-identified prior to analysis. Other efforts to maintain the confidentiality of participants included referring to specimens and other records via an assigned, coded identification number.

An overview of the characteristics of donors with acute COVID-19 is provided in **Table S1**. Similar to convalescent donors, the majority of donors with acute COVID-19 (67%) had a known sick contact with COVID-19 or suspected exposure to SARS-CoV-2 (**Table S1**). Similar to the convalescent donors, commonly reported symptoms included dyspnea, cough, fatigue, fever, and anosmia; though dyspnea was more common in donors with acute COVID-19 (**Table S1**). Peak disease severity and disease severity at the time of blood collection was ranked using a score from 0-10 based on a modified version of the ordinal scale defined in the preliminary report for the ACTT-1 study (Beigel et al., 2020), as in **Table S2** (see below). Seventy-one percent of donors experienced severe or critical illness (**Table S1**). Of note, 87% of donors with acute COVID-19 had at least one known underlying medical condition (**Table S1**). In the case of 2 acute donors, a second sample was obtained on or prior to day 15 PSO and while the donor was still exhibiting symptoms consistent with acute COVID-19. Both samples were included in ADIM and correlogram analyses. Thus, while 24 distinct acute subjects were enrolled in this study, 26 subjects were considered for ADIM scoring and correlogram analyses.

##### Disease Severity Scoring System

Given that the WHO Clinical Management of COVID-19 Interim Guidance document was last updated May 27, 2020, and is a descriptive scoring system without numerical categories, a new scoring system was developed for this study. This scoring system is described in detail in the supplementary materials in **Table S2**. It was developed and applied by an Infectious Diseases physician following review of the scoring systems in use in contemporary COVID-19 literature. This scoring system is based on the NIH ordinal scale (Beigel et al., 2020). Category “0” was added to this ordinal scale to account for healthy unexposed control donors and convalescent COVID-19 donors who had fully recovered by the time of blood donation. Additionally, category “1” was added to the scale to account for individuals with subclinical or asymptomatic infection. As the Beigel et al., 2020, study was focused on acute, symptomatic disease in a hospitalized population, they likely did not have a need to include equivalent categories. Category “8” was added to the scoring system presented in this publication in order to differentiate between those requiring ICU level care for critical illness for reasons other than mechanical ventilation or extracorporeal membrane oxygenation (ECMO) for acute respiratory distress syndrome (ARDS) or other respiratory failure (e.g., the use of inotropes/vasopressors for blood pressure support in the setting of hypotension, or continuous renal replacement therapy in the setting of acute renal failure).

### Method Details

#### PBMC and plasma isolation and handling

Whole blood was collected in either EDTA (most acute disease) or acid citrate dextrose (ACD; 2 acute and all convalescent donors) tubes (BD vacutainer tubes, Franklin Lakes, NJ, USA) and stored at room temperature prior to processing for PBMC isolation. For healthy, unexposed donor samples, whole blood was collected in a heparin coated blood bag. Whole blood was processed as previously described (Grifoni et al., 2020a). In brief, PBMC were isolated by density-gradient sedimentation, as described below. Sterile technique was used to transfer the blood to conical tubes. Whole blood was diluted 1:2 in room temperature RPMI (Corning, Manassas, VA, USA), then layered over an appropriate volume of room temperature Histopaque (Histopaque-1077 Cell Separation Medium, Sigma-Aldrich, St. Louis, MO, USA) or Ficoll-Paque (Lymphoprep, Nycomed Pharma, Oslo, Norway), then centrifuged for 25 min at 1850 rpm at room temperature with no brake to separate the cellular fraction and plasma. The plasma was then carefully removed, aliquoted, and stored at −80°C. The PBMC buffy coat was then collected and washed with RPMI. If red blood cell contamination was present, red blood cells were lysed using ACK Lysing Buffer (GIBCO, Grand Island, NY, USA). Lysis was stopped by the addition of an equal volume of R10 medium (RPMI with 10% FBS, 1% GlutaMAX, 1% penicillin-streptomycin) followed by centrifugation for 7-10 min at 1800 rpm at 4°C. An aliquot of cells was placed in BD Cytofix fixation buffer (BD, Franklin Lakes, NJ, USA) or 1% formaldehyde and cells were counted manually using a hemocytometer or using a BD Accuri flow cytometer (BD, Franklin Lakes, NJ, USA). Isolated PBMC were then cryopreserved in cell recovery medium containing 10% DMSO (GIBCO) and 90% heat inactivated fetal bovine serum (FBS; Hyclone Laboratories, Logan UT), placed in a Mr. Frosty freezing container (Thermo Scientific, USA) and frozen overnight at −80°C, and then transferred to liquid nitrogen for further storage until used in the assays.

All blood samples and blood products were handled in a BSL-2 laboratory with the use of appropriate personal protective equipment and safety precautions, in accordance with the blood processing protocol approved by the LJI Institutional Biosafety Committee (BHR15-SC). Where appropriate, plasma was heat inactivated for 30 min at 54-56°C prior to use.

#### Serum isolation and handling

Whole blood was collected in serum separator tubes (SST BD vacutainer tubes, Franklin Lakes, NJ, USA) for serum isolation. SSTs were centrifuged for 4 min at 1100 rcf at 4°C. The serum was then removed from the upper portion of the tube, aliquoted, and stored at −80°C. Where appropriate, serum was heat inactivated for 30 min at 54-56°C prior to use.

#### SARS-CoV-2 RT-qPCR

To further ensure laboratory safety and support previous findings that infectious SARS-CoV-2 virus cannot be isolated from blood, we performed RT-qPCR to assess SARS-CoV-2 viral RNA in human blood samples. Viral RNA was isolated from plasma, serum, or PBMC using Quick-RNA Viral Kit (Zymo Research). RT-qPCR was performed using TaqMan Fast Virus 1-Step Master Mix (Applied Biosystems) with CDC RUO primers and probes targeting the SARS-CoV-2 N1 gene (nCOV_N1 Forward Primer Aliquot, nCOV_N1 Reverse Primer Aliquot, and nCOV_N1 Probe Aliquot; Integrated DNA Technologies) and CFX96 Real-Time PCR Detection System (Bio-Rad) (https://www.cdc.gov/coronavirus/2019-ncov/lab/rt-pcr-panel-primer-probes.html). The SARS-CoV-2 plasmid (Integrated DNA Technologies) was used as a standard and control. Plasma for each donor was tested for SARS-CoV-2 viral RNA before PBMC sorting from the same donor. All healthy controls and convalescent plasma were viral RNA negative. All acute plasma samples were viral RNA negative, except one acute plasma sample. This was determined to be positive (< 9,000 RNA copies / mL plasma; Ct = 34.9), but considered to have a concentration of viral RNA well below the level necessary for isolation of infectious virus (Wölfel et al., 2020).

#### SARS-CoV-2 ELISAs

Recombinant SARS-CoV-2 Receptor Binding Domain (RBD) protein and Spike protein were obtained from the Saphire lab. This RBD sequence is 346 amino acids in length, corresponding to amino acids 319-591 of the Spike protein sequences in the Protein Data Bank (PDB) deposited by Premkumar et al., 2020 (PDB 6VSB) and for the reference strain Wuhan-Hu-1 (PDB 6XR8). Recombinant Nucleocapsid protein was obtained from GenScript (Z03488). Corning 96-well half-area plates (ThermoFisher 3690) were coated with 1μg/mL SARS-CoV-2 antigen overnight at 4°C. The ELISA protocol has been previously described (Grifoni et al., 2020a). Briefly, the following day, plates were blocked with 3% milk (Skim Milk Powder ThermoFisher LP0031 by weight/volume) in Phosphate Buffered Saline (PBS) containing 0.05% Tween-20 (ThermoScientific J260605-AP) for 1.5 h at room temperature. Plasma was heat inactivated at 56°C for 30-60 min and then diluted in 1% milk in 0.05% PBS-Tween 20 starting at a 1:3 dilution followed by serial dilutions by 3. Plasma was incubated for 1.5 h at room temperature. Plates were washed 5 times with 0.05% PBS-Tween 20. Secondary antibodies were diluted in 1% milk in 0.05% Tween-20 and incubated for 1 h. For IgG, anti-human IgG peroxidase antibody produced in goat (Sigma A6029) was used at a 1:5,000 dilution. Anti-human IgG peroxidase antibody from Sigma was tested and found comparable to anti-human IgG Fc Pan peroxidase (Hybridoma 6043HRP). For IgM, anti-human IgM peroxidase antibody produced in goat (Sigma A6907) was used at a 1:10,000 dilution. For IgA, anti-human IgA horseradish peroxidase antibody (Hybridoma Reagent Laboratory HP6123-HRP) was used at a 1:1,000 dilution. Plates were washed 5 times with 0.05% PBS-Tween-20. Plates were developed with TMB Substrate Kit (ThermoScientific 34021) for 15 min at room temperature. The reaction was stopped with 2M sulfuric acid. Plates were read on a Spectramax Plate Reader at 450 nm using SoftMax Pro. Endpoint titers were plotted for each specimen, using background subtracted data. A positive control standard was created by pooling plasma from 6 convalescent COVID-19 donors to normalize between experiments. A Mann-Whitney analysis was done to compare endpoint titers between COVID-19 and negative specimens.

#### Neutralizing Antibody Assays

The live neutralizing antibody assay was performed at The University of North Carolina, Chapel Hill, as previously described (Premkumar et al., 2020). The pseudovirus neutralizing antibody assay was performed by the Saphire laboratory. Plasmids for full-length SARS-Cov-2 S were generated from synthetic codon-optimized DNA (Wuhan-Hu-1 isolate, GenBank: MN908947.3) through sub-cloning into the pHCMV3 expression vector, with a stop codon included prior to the HA tag. Positive clones were fully sequenced to ensure that no additional mutations were introduced. Recombinant SARS-CoV-2-pseduotyped VSV-DG-GFP were generated by transfecting 293T cells with phCMV3-SARS-CoV-2 S using TransIT-293 Transfection Reagent (Mirus Bio) according to the manufacturer’s instructions. At 24 h post-transfection, cells were washed 2x with OptiMEM and were infected with rVSV-G pseudotyped DG-GFP parent virus (VSV-G^∗^DG-GFP) at MOI = 2 for 2 h with rocking. The virus was then removed, and the cells were washed twice with OPTI-MEM containing 2% FBS (OPTI-2) before fresh OPTI-2 was added. Supernatants containing rVSV-SARS-2 were removed 24 h post-infection and clarified by centrifugation. Vero cells were seeded in 96-well plates at a density sufficient to produce a monolayer at the time of infection. Then, 10-fold serial dilutions of pseudovirus were made and added to cells in triplicate wells. Infection was allowed to proceed for 12-16 h at 37þC. The cells were then fixed with 4% PFA, washed two times with 1xPBS and stained with Hoescht (1ug/mL in PBS). After two additional washes with PBS, pseudovirus titers were quantified as the number of focus forming units (ffu/mL) using a CellInsight CX5 imager (ThermoScientific) and automated enumeration of cells expressing GFP. Pre-titrated amounts of rVSV-SARS-CoV-2 was incubated with serially diluted human sera or plasma at 37°C for 1 h before addition to confluent Vero monolayers in 96-well plates. Infection proceeded for 12-16 h at 37°C in 5% CO_2_ before cells were fixed in 4% paraformaldehyde and stained with 1ug/mL Hoescht. Cells were imaged using a CellInsight CX5 imager and infection was quantitated by automated enumeration of total cells and those expressing GFP. Infection was normalized to the average number of cells infected with rVSV-SARS-CoV-2 incubated with normal human sera. Limit of detection (LOD) was established as < 1:20. Data are presented as the relative infection for each concentration of sera. Neutralization IC_50_ titers were calculated using “One-Site Fit LogIC50” regression in GraphPad Prism 8.0.

#### Flow Cytometry

##### T cell stimulation

For all flow cytometry assays of stimulated T cells, cryopreserved cells were thawed by diluting them in 10mL pre-warmed complete RPMI containing 5% human AB serum (Gemini Bioproducts) in the presence of benzonase (20ul/10mL) and spun at 1200 rpm for 7 min. Supernatants were carefully removed by pipetting and cells were resuspended in warm medium, counted and apportioned for assays.

##### Direct ex vivo PBMC immune cell phenotyping

For the surface stain, 1x10^6^ PBMCs were resuspended in 100 ul PBS with 2% FBS (FACS buffer) and incubated with BD human FC block (BD Biosciences, San Diego, CA) for 10 min at room temperature (RT). Without washing, fluorescently-labeled chemokine receptor antibodies were added to cells and incubated at 37°C in the dark for 10 min. Antibody mix containing the rest of the surface antibodies were then added directly to cells and incubated for 20 min at RT in the dark. Following surface staining, cells were washed once with FACS buffer and resuspended in 100 uL BD fixation/permeabilization solution (BD Biosciences, San Diego, CA) and incubated at 4°C for 45 min in the dark. Cells were then washed twice with permeabilization buffer and stained with intracellular and intranuclear antibodies for 20 min at 4°C in the dark. After staining, cells were washed once with permeabilization buffer and resuspended in FACS buffer. All samples were acquired on a BD FACSymphony S6 cell sorter. A list of antibodies used in this panel can be found in **Table S3**.

##### Activation induced cell marker assay

Assays were conducted as previously described (Grifoni et al., 2020a; Morou et al., 2019; Reiss et al., 2017). Cells were cultured for 24 h in the presence of SARS-CoV-2-specific MPs [1 ug/mL] in 96-well U bottom plates at 1x10^6^ PBMC per well in complete RPMI containing 5% Human AB Serum (Gemini Bioproducts). Prior to addition of peptide MPs, cells were blocked at 37°C for 15 min with 0.5ug/mL anti-CD40 mAb (Miltenyi Biotec). A stimulation with an equimolar amount of DMSO was performed as negative control, Staphylococcal enterotoxin B (SEB, 1 ug/mL) and stimulation with a combined CD4 and CD8 cytomegalovirus MP (CMV, 1 ug/mL) were included as positive controls. Supernatants were harvested at 24 h post-stimulation for multiplex detection of cytokines. Antibodies used in the AIM assay are listed in **Table S4**. AIM^+^ gates were drawn relative to the unstimulated condition for each donor.

##### Intracellular cytokine staining assay

For the intracellular cytokine staining, PBMC were cultured in the presence of SARS-CoV-2-specific MPs [1 ug/mL] for 9 h at 37°C. Golgi-Plug containing brefeldin A (BD Biosciences, San Diego, CA) and monensin (Biolegend, San Diego, CA) were added 3 h into the culture. Prior to addition of peptide MPs, cells were blocked at 37°C for 15 min with 0.5ug/mL anti-CD40 mAb (Miltenyi Biotec). Cells were then washed and surface stained for 30 min on ice, fixed with 1% of paraformaldehyde (Sigma-Aldrich, St. Louis, MO) and kept at 4°C overnight. Antibodies used in the ICS assay are listed in Table S4. The gates applied for the identification of CD40L^+^, CD40L^+^IFNg^+^, CD40L^+^IL-2^+^, CD40L^+^IL-10^+^, CD40L^+^IL-17^+^ or CD40L^+^TNFa^+^ production on non-CD45RA^+^CCR7^+^ CD4^+^ T cells were defined according to the cells cultured with DMSO for each individual. The gates applied for the identification of IFNg^+^, IFNg^+^TNFa^+^ or IFNg^+^GzmB^+^ production on non-CD45RA^+^CCR7^+^ CD8^+^ T cells were defined according to the cells cultured with DMSO for each individual. Antibodies used in the ICS assay are listed in **Table S5**.

#### Cytokine bead assays

The human anti-virus response panel (13-plex)(BioLegend, Cat No:740349) was used to quantitate human plasma cytokines (IL-1b, IL-6, IL-8, IL-10, IL-12p70, IFN-a2, IFN-b, IFN-l1, IFN-l2/3, IFNg, TNFa, IP-10, GM-CSF). The human T helper cytokine panel (IL-2, IL-4, IL-5, IL-6, IL-9, IL-10, IL-13, IL-17A, IL-17F, IL-21, IL-22, IFNg and TNFa) was run for AIM supernatants (BioLegend, Cat No:740721). Plasma samples were freshly thawed and run in a 2-fold dilution in matrix B per the manufacturer’s instructions. Samples were not heat inactivated and centrifuged per recommendation of the directions before the assay was run (1000 x g for 5 min). AIM samples were freshly thawed, centrifuged (1000 x g for 5 min), and run undiluted in assay buffer per manufacturer recommendations. The recommended filter plate method was used, and all steps were followed per the manufacturer’s protocol. Samples were acquired on a Canto II flow cytometer (BD) using a high throughput sampler. Samples were run in duplicate, and standards run on all plates. CST was run prior to all runs to ensure low detector CVs and set laser delay.

#### Correlation plots and heatmap visualizations

Correlograms plotting the Spearman rank correlation coefficient (r), between all parameter pairs were created with the corrplot package (v0.84) (Wei and Sikmo, 2017) running under R (v3.6.1) in Rstudio (1.1.456). Clustering of parameters was performed using the ‘hclust’ option of corrMatOrder on the acute and convalescent samples combined. Parameters with low information content were removed (predominantly negative or ties) prior to graphing. “Peak disease” parameter was fixed as the bottom row for easy visualization. Spearman rank two-tailed P values were calculated using corr.test (psych v1.8.12) and graphed (ggplot2 v3.1.1) based on ^∗^ p < 0.05, ^∗∗^p < 0.01, ^∗∗∗^ p < 0.001. The same parameter ordering was then used throughout all full correlograms. Small correlograms were made based on the same data, but with curated parameters and ordering (**Figure S7**). For the correlograms in Figure S7, false discover rate (FDR) corrections were performed using the Benjamini-Hochberg test at an FDR < 0.05 significance threshold. Correlogram parameter labels were shortened throughout the text for clarity. In all correlograms, CD4^+^ and CD8^+^ T cell values used for analyses are either the percentage of total (T) CD4^+^ or CD8^+^ or the percentage of non-naive (NN) CD4^+^ or CD8^+^, where naive T cells are defined as CCR7^+^CD45RA^+^. NK cells are defined as CD56^dim^CD16^+^CD3^-^CD19^-^ cells and are calculated as the frequency out of total PBMC. Total B cells are CD19^+^CD3^-^ PBMCs. Naive B cells are CD19^+^CD27^-^IgD^+^. Comorbidities is the sum of any pre-existing conditions per patient assessed at time of enrollment in the study. Contemp. Disease (contemporaneous disease score) is the disease score assigned to the donor at the time of sample collection. Peak disease (peak disease score) is the disease score assigned to the donor at maximum disease severity. All cytokines reported were measured in pg/mL; e.g., “IFNg CD8A/B” is pg/mL IFNg in culture supernatants after stimulation of PBMCs with the SARS-CoV-2 CD8-A/B peptide megapool, data shown in Figure 3. ICS data used are the percentage of SARS-CoV-2 antigen-specific cytokine-producing CD4^+^ or CD8^+^ cells out of non-naive CD4^+^ or CD8^+^ T cells respectively in response to SARS-CoV-2 antigens, shown in Figures 2 & 3. Cytokines measured in AIM supernatants are listed as the cytokine measured in response to a specific SARS-CoV-2 peptide megapool in pg/mL, e.g., IL-4 S. The column labeled “CD4:CD8” refers to the ratio of the percentages of total CD4^+^ T cell to total CD8^+^ T cell gated out of PBMC. A complete list of all data used for correlation analyses can be found in **Data S1**.

### Quantification and Statistical Analysis

Data and statistical analyses were done in FlowJo 10 and GraphPad Prism 8.4, unless otherwise stated. The statistical details of the experiments are provided in the respective figure legends. Data plotted in linear scale were expressed as mean + standard deviation (SD). Data plotted in logarithmic scales were expressed as geometric mean + geometric standard deviation (SD). Correlation analyses were performed using Spearman, while Mann-Whitney or Wilcoxon tests were applied for unpaired or paired comparisons, respectively. Correlogram statistical analyses are described above. Details pertaining to significance are also noted in the respective legends. T cell data have been calculated as background subtracted data or stimulation index. Background subtracted data were derived by subtracting the percentage of AIM^+^ cells after SARS-CoV-2 stimulation from the DMSO stimulation. If the AIM^+^ cells percentage after DMSO stimulation was equal to 0, the minimum value across each cohort was used. When two stimuli were combined together, the percentage of AIM^+^ cells after SARS-CoV-2 stimulation was combined and either subtracted twice or divided by twice the value of the percentage of AIM^+^ cells derived from DMSO stimulation. Additional data analysis techniques are described in the Methods sections above.

## Web Resources

US Census Bureau QuickFacts: San Diego County, California; California, https://www.census.gov/quickfacts/fact/table/sandiegocountycalifornia, CA/PST045219
WHO Coronavirus Disease (COVID-19) Dashboard, https://covid19.who.int

## Supplemental Information

Document S1. Tables S1-S5

Data S1. Compiled Raw Data For Correlation Analyses, Related to Figures 4–7
