# Supplementary material for: Antigen-Specific Adaptive Immunity to SARS-CoV-2 in Acute COVID-19 and Associations with Age and Disease Severity
Source: Cell. 2020 Nov 12;183(4):996–1012.e19. doi: 10.1016/j.cell.2020.09.038 (PMC7494270; doi:10.1016/j.cell.2020.09.038)
Supplement: Document S1. Tables S1-S5 [file mmc1.pdf]

Cell, Volume *183*

Supplemental Information

Antigen-Specific Adaptive Immunity to SARS-CoV-2 in Acute COVID-19 and Associations with Age and Disease Severity

Carolyn Rydyznski Moderbacher, Sydney I. Ramirez, Jennifer M. Dan, Alba Grifoni, Kathryn M. Hastie, Daniela Weiskopf, Simon Belanger, Robert K. Abbott, Christina Kim, Jinyong Choi, Yu Kato, Eleanor G. Crotty, Cheryl Kim, Stephen A. Rawlings, Jose Mateus, Long Ping Victor Tse, April Frazier, Ralph Baric, Bjoern Peters, Jason Greenbaum, Erica Ollmann Saphire, Davey M. Smith, Alessandro Sette, and Shane Crotty

Table S1. Participant characteristics, Related to Fig.1-7

	Unexposed (n = 15)	Convalescent COVID-19 (n = 15)	Acute COVID-19 (n = 24)
**Age (years)**	20-64 [Median = 26, IQR = 27.5]	27-68 [Median = 44, IQR = 11.5]	25-86 [Median = 55.5, IQR = 33]
**Gender**			
**Male (%)**	20% (3/15)	47% (7/15)	71% (17/24)
**Female (%)**	80% (12/15)	53% (8/15)	29% (7/24)
**Race**			
**African American or Black (%)**	0% (0/15)	N/A	13% (3/24)
**Alaskan Native or American Indian (%)**	0% (0/15)	N/A	0% (0/24)
**Asian (%)**	13% (2/15)	N/A	4% (1/24)
**Native Hawaiian or Pacific Islander (%)**	7% (1/15)	N/A	0% (0/24)
**Other/Mixed Race (%)**	7% (1/15)	N/A	46% (11/24)
**Unknown (%)**	33% (5/15)	100% (15/15)	8% (2/24)
**White (%)**	40% (6/15)	N/A	29% (7/24)
**Ethnicity**			
**Hispanic (%)**	20% (3/15)	N/A	29% (7/24)
**Non-Hispanic (%)**	60% (9/15)	N/A	58% (14/24)
**Unknown (%)**	20% (3/15)	100% (15/15)	13% (3/24)
**Hospitalization status**			
**Never hospitalized (%)**	100% (15/15)	100% (15/15)	8% (2/24)
**Hospitalized at the time of blood collection (%)**	0% (0/15)	0% (0/15)	92% (22/24)
**Sample Collection Dates**	September 2018-October 2019	March-May 2020	March-June 2020
**SARS-CoV-2 PCR Positivity**	N/A	100% (13/13); 13 tested	100% (23/23); 23 tested
**Antibody Test Positivity**	N/A	100% (15/15)	75% (3/4); 4 tested
**Peak Disease Severity**			
**Healthy/No disease (0)**	100% (15/15)	0% (0/15)	0% (0/24)
**Mild (1-2)**	N/A	73% (11/15)	8% (2/24)
**Moderate (3-5)**	N/A	27% (4/15)	13% (3/24)
**Severe (6-7)**	N/A	0% (0/15)	33% (8/24)
**Critical (8-9)**	N/A	0% (0/15)	38% (9/24)
**Fatal (10)**	N/A	0% (0/15)	8% (2/24)
**Disease Severity at Time of Blood Collection**			***n=30 (4 donors with 2 time points, 1 donor with 3 time points)**
**Healthy/No disease (0)**	100% (15/15)	100% (15/15)	0% (0/30)
**Mild (1-2)**	N/A	0% (0/15)	7% (2/30)
**Moderate (3-5)**	N/A	0% (0/15)	33% (10/30)
**Severe (6-7)**	N/A	0% (0/15)	37% (11/30)
**Critical (8-9)**	N/A	0% (0/15)	23% (7/30)
**Fatal (10)**	N/A	0% (0/15)	0% (0/30)
**Presenting Symptoms**			
**Cough (%)**	N/A	80% (12/15)	67% (16/24)
**Fatigue (%)**	N/A	73% (11/15)	25% (6/24)
**Fever (%)**	N/A	47% (7/15)	54% (13/24)
**Anosmia (%)**	N/A	40% (6/15)	21% (5/24)
**Dyspnea (%)**	N/A	47% (7/15)	79% (19/24)
**Diarrhea (%)**	N/A	13% (2/15)	17% (4/24)
**Days Post Symptom Onset at Collection**	N/A	25-56 [Median = 34, IQR = 16]	4-37 [Median = 11, IQR = 11.5]; *n=30 (4 donors with 2 time points, 1 with 3 time points)
**Past Medical History**			
**No known (%)**	100% (15/15)	53% (8/15)	13% (3/24)
**Hyperlipidemia (%)**	N/A	20% (3/15)	8% (2/24)
**Hypertension (%)**	N/A	20% (3/15)	29% (7/24)
**Coronary artery disease (%)**	N/A	7% (1/15)	8% (2/24)
**Diabetes (%)**	N/A	7% (1/15)	17% (4/24)
**Obesity (%)**	N/A	7% (1/15)	17% (4/24)
**Other (%)**	N/A	40% (6/15)	63% (15/24)
**Known or suspected sick contact/exposure (%)**	0% (0/15)	87% (13/15)	67% (16/24)

Table S2. COVID-19 disease severity classifications, Related to Fig. 4-7

Disease Severity Score (0-10)	Description	Hospitalization Requirement	Supplemental Oxygen Needs for COVID-19
**Healthy**			
**0**	No known COVID-19 diagnosis or convalescent (fully recovered from COVID-19 and 3 weeks or more from initial diagnosis, without symptoms)	No	None
**Mild**			
**1**	Subclinical infection or asymptomatic (no symptoms preceding or up to time of COVID-19 diagnosis)	No	None
**2**	Symptoms consistent with COVID-19 without limitation of activities	No	None
**Moderate**			
**3**	Symptoms consistent with COVID-19 that limit activities and/or with home oxygen requirement (above baseline)	No	+/- home supplemental oxygen (above baseline)
**4**	Hospitalized for a reason other than COVID-19 but incidentally found to be positive on testing or recovered from COVID-19 to the extent that hospital care is no longer required for COVID-19 but hospitalization extended for infection-control or other reasons unrelated to COVID-19 management	Yes, but not requiring (ongoing) hospital care for COVID-19	None
**5**	Requiring ongoing medical care for COVID-19 other than supplemental oxygen (non-ICU level care)	Yes (non-ICU)	None
**Severe**			
**6**	Requiring ongoing medical care for COVID-19 including supplemental oxygen via nasal cannula	Yes (non-ICU)	Nasal cannula
**7**	Requiring ongoing medical care for COVID-19 including high-flow supplemental oxygen or non-invasive ventilation	Yes (non-ICU)	High-flow oxygen delivery device or non-invasive ventilation
**Critical**			
**8**	Requiring vasopressor support or other ICU level care for COVID-19 but not mechanical ventilation or extracorporeal membrane oxygenation (ECMO)	Yes (ICU)	+/- but not requiring mechanical ventilation or ECMO
**9**	Requiring ICU level care including mechanical ventilation or ECMO for management of COVID-19	Yes (ICU)	Mechanical ventilation or ECMO
**Fatal**			
**10**	Death attributable to COVID-19	+/-	+/-

Table S3. Immune cell phenotyping panel, Related to Fig. 5-7

Marker-Fluorophore	Clone	Vendor	Catalog #
KI67-FITC	B56	BD	556026
CD4-percp efluor710	SK3	Invitrogen	46-0047-42
Gzm B- af647	GB11	Biolegend	515406
IgM- af700	MHM-88	Biolegend	314538
CD27-APCcy7	O323	Biolegend	302816
IgD- PE	IA6-2	BD	555779
CD56- PE Dazzle	HCD56	Biolegend	318348
HLA-DR	Tu39	BD	746979
CD19 PE-Cy5	HIB19	Biolegend	302210
CD38- PECy7	HIT2	Invitrogen	25-0389-42
CXCR5-bv421	J252D4	Biolegend	356920
CD14- bv510	63D3	Biolegend	367123
CD45RA- bv570	HI100	Biolegend	304132
CXCR3- bv605	G025H7	Biolegend	353728
CD20- bv650	2H7	Biolegend	302336
CCR7-bv711	G043H7	Biolegend	353228
PD-1-bv786	EH12.2H7	Biolegend	329930
CD3- buv395	UCHT1	BD	563546
Fixable live/dead Blue	N/A	Thermo Fisher	L34962
CCR6-buv496	11A9	BD	612948
CD16-buv737	3G8	BD	612786
CD8- buv805	SK1	BD	612889

Table S4. AIM Panel, Related to Fig. 2-7

Marker-Fluorophore	Clone	Vendor	Catalog #
CD69-FITC	FN50	Biolegend	310904
CD4-percp efluor710	SK3	Invitrogen	46-0047-42
Ox40-APC	Ber-Act35	Biolegend	350008
CD14/CD16/CD20-APCcy7	61D3/eBioCB16/2H7	Invitrogen	47-0149-42/47-0168-42/47-0209-42
PD-L1- PE	29E.2A3	Biolegend	329706
CD40L	24-31	Biolegend	310840
CD38- PECy7	HIT2	Invitrogen	25-0389-42
CXCR5-bv421	J252D4	Biolegend	356920
CD25-bv510	BC96	Biolegend	302640
CD45RA- bv570	HI100	Biolegend	304132
CXCR3- bv605	G025H7	Biolegend	353728
CCR7-bv711	G043H7	Biolegend	353228
PD-1-bv786	EH12.2H7	Biolegend	329930
CD3- buv395	UCHT1	BD	563546
Fixable live/dead Blue	N/A	Thermo Fisher	L34962
CCR6-buv496	11A9	BD	612948
ICOS-buv563	DX29	BD	741421

Table S5. ICS panel, Related to Fig. 2,3,5,6,7

Marker-Fluorophore	Clone	Vendor	Catalog #
IL13-FITC	85BRD	Invitrogen	11-7136-42
CD4-percp efluor710	SK3	Invitrogen	46-0047-42
GzmB-alexa-fluor 647	GB11	Biolegend	515406
IL2- alexa-fluor 700	MQ1-17H12	Biolegend	500320
CD14/CD16/CD20-APCcy7	61D3/eBioCB16/2H7	Invitrogen	47-0149-42/47-0168-42/47-0209-42
CD40L - PE	24-31	Biolegend	310806
IL10-PE-Dazzle	JES3-19F1	Biolegend	506812
TNFa- PE-Cy7	Mab11	Invitrogen	25-7349-82
CXCR5-bv421	J252D4	Biolegend	356920
CD45RA- bv570	HI100	Biolegend	304132
CXCR3- bv605	G025H7	Biolegend	353728
CCR7-bv711	G043H7	Biolegend	353228
IL17a-bv786	BL168	Biolegend	512338
CD3- buv395	UCHT1	BD	563546
Fixable live/dead Blue	N/A	Thermo Fisher	L34962
IFNg- buv737	4S.D3	BD	612845
CD8-buv805	SK1	BD	612889
